# Language of instruction in schools in low‐ and middle‐income countries: A systematic review

**DOI:** 10.1002/cl2.1351

**Published:** 2023-10-03

**Authors:** Pooja Nakamura, Adria Molotsky, Rosa Castro Zarzur, Varsha Ranjit, Yasmina Haddad, Thomas De Hoop

**Affiliations:** ^1^ International Development Division American Institutes for Research Arlington Virginia USA

## Abstract

Based on the theory of change, we gather, organize, and synthesize the evidence on the impact of three language of instruction (LOI) choices (teaching in mother tongue [MT] with later transition, teaching in a non‐MT language, or teaching in two or more languages at one time) on literacy and biliteracy outcomes. We focus on quantitative and qualitative studies of LOI interventions in low‐ and middle‐income countries (LMICs) and consider languages that are commonly spoken in the developing world. As such, we include studies that examine transfers from local languages to English, but not those evaluating transfers from local languages to languages that are less spoken in LMICs (e.g., Swedish).

## 1 PLAIN LANGUAGE SUMMARY

### 1.1 Language of instruction at school: Supporting mother tongue teaching vs later acquired language teaching

While there are numerous studies providing evidence for the use of mother tongue (MT) instruction for reading outcomes, there are only a limited number of studies discussing the evidence on the impact of language of instruction (LOI) transition policies on biliteracy and multilingual literacy outcomes. This study is the first systematic review of this evidence.

### 1.2 What is this review about?

Over the last 50 years, schooling has expanded dramatically in most low‐ and middle‐income countries (LMICs). However, while children are in school more than ever before, a large proportion of students are not acquiring basic literacy and numeracy skills. Although a myriad of factors contributes to this state of learning poverty, the role of language is essential as all learning happens in and through language.

LOI policies focus on the mandated language teachers should use when teaching students in the classroom. This review looked at whether MT‐based LOI and language transition policies facilitated reading and biliteracy and multilingual literacy outcomes for students and whether these policies have different effects on skill development by language group.

**What is the aim of this review**

This Campbell systematic review examines the effects of language of instruction policies on students’ literacy, biliteracy and multilingual literacy skill development in low‐ and middle‐income countries.

### 1.3 What studies are included?

The review summarizes evidence from 45 high‐quality studies, including 11 randomized controlled trials, 11 quasi‐experimental studies, seven cross‐sectional studies, and 16 qualitative studies.

The included studies evaluate the effects of MT‐based LOI and LOI transition policies on students’ biliteracy and multilingual literacy skill development. The studies spanned the period from 1995 to 2020 and were carried out in sub‐Saharan Africa and South‐ and Southeast Asia.

### 1.4 Does prescribing mother tongue‐based instruction in primary school lead to improved literacy and biliteracy outcomes for students in bilingual and multilingual contexts?

Meta‐analyses and quantitative narrative syntheses indicate that MT‐based LOI interventions may improve students’ letter knowledge, word reading, sentence reading, and reading comprehension in the students’ MT, improve students’ word and sentence reading, and reading comprehension in the national language, and improve students’ oral language proficiency, word, and sentence reading, reading comprehension, and writing in the later acquired language.

It is still unclear to what extent MT instruction can support English (or a later acquired language) compared to investments in high quality teaching in the later acquired language alone.

Furthermore, the systematic review revealed an evidence gap on how MT‐based programs may impact later language literacy acquisition.

### 1.5 What factors affect how well mother tongue‐based language of instruction policies work for biliteracy outcomes?

The qualitative studies suggest that high‐quality teaching and learning materials in the MT coupled with improved curriculum and bilingual materials throughout the classroom are necessary for a successful MT‐based LOI program.

These programs overwhelmingly received positive reception and wide support by students and teachers alike as students and parents perceive that these programs improve teaching quality, increase student motivation in the classroom, and respondents report improvements in bilingual reading skills.

### 1.6 What do the findings of this review mean?

MT‐based LOI policies are likely to positively affect students’ literacy outcomes in their MT, but the evidence base is small and restricted to more costly interventions.

The evidence is still inconclusive on how much MT instruction can support English (or later acquired language) relative to high quality teaching in the later acquired language. There is an evidence gap on the impact of MT‐based policies on transition and later language outcomes.

The findings from a limited quantitative evidence base suggest that high‐quality MT interventions may lead to gains in MT reading outcomes. Qualitative results showed that students felt that their English improved in programs for English as a later acquired language.

Given the lack of conclusive evidence on the trade‐off between supporting high‐quality English LOI versus high quality MT LOI with a later transition to English‐medium education, more research is needed.

### 1.7 How up‐to‐date is this review?

The review authors searched for studies up to 2020.

## 2 EXECUTIVE SUMMARY

### 2.1 Background

Learning poverty—defined as the share of 10‐years‐olds in low‐ and middle‐income countries (LMICs) who cannot read a simple text—was 53% before the Covid‐19 pandemic and is expected to reach 70% by 2023 (World Bank, 2021). Although a myriad of factors contribute to learning poverty, language is a critical one. On average, nearly 40% of students in LMICs are educated in languages they do not speak or understand (up to 90% in some countries) (World Bank, 2021). Furthermore, in nearly all LMICs, students need to transition to a new language of instruction (LOI) toward the end of primary school, despite very little evidence of when and how that transition should be made.

There is now substantial evidence that mother tongue (MT)‐based multilingual education (i.e., the child is taught in their own home language first) has multiple benefits (Collier & Thomas, 2017; Evans & Acosta, 2020; Nag, Vagh, Dulay, & Snowling, 2019). At the same time, there is a well‐established link between skills in a postcolonial language and socioeconomic mobility leading to high demand for earlier and earlier introduction to international post‐colonial languages in schools (Azam, Chin, & Prakash, 2010; Coleman, 2011). Other factors that complicate LOI choices include linguistically heterogenous classrooms, lack of teaching and learning materials, limited trained teachers, and lack of political or community will (Piper, Zuilkowski, & Ong'ele, 2016; Trudell & Piper, 2013).

This systematic review focuses on reconciling these evidence gaps by examining the impact of LOI choices—especially MT education as well as the timing of transition to a new LOI—on primary school literacy outcomes in LMICs.

### 2.2 Objectives

Based on the theory of change, we gather, organize, and synthesize the evidence on the impact of three LOI choices (teaching in MT with later transition, teaching in a non‐MT language, or teaching in two or more languages at one time) on literacy and biliteracy outcomes. We focus on quantitative and qualitative studies of LOI interventions in LMICs and consider languages that are commonly spoken in the developing world. As such, we include studies that examine transfers from local languages to English, but not those evaluating transfers from local languages to languages that are less spoken in LMICs (e.g., Swedish).

This study aims to answer the following research questions:

*Primary research questions*:

1. What are the short‐ and long‐term impacts of **initial LOI** choices on literacy and biliteracy outcomes, and how do they differ across various LMIC contexts?
2. What are the short‐ and long‐term impacts of **LOI transition** on literacy and biliteracy outcomes, and how do they differ across various LMIC contexts?
3. What qualitative factors support effective implementation of LOI transition policies and practices?

*Secondary research question*:

4. What are the evidence gaps about the role of LOI choices in bilingual and multilingual educational contexts in LMICs?

### 2.3 Selection criteria

We consider studies with interventions comprising LOI choices made by educational policies or programs that *directly* aim to increase children's literacy in bilingual or multilingual LMIC education contexts. These include early learning programs for MT education, official LOI policies, teacher training for MT programs or bilingual or multilingual education programs, technology‐based interventions for MT or bilingual or multilingual education programs, among other interventions.

We limited this systematic review to studies that had interventions for primary and secondary school‐aged children in LMICs. We included studies about the effects of LOI choices regardless of the educational status or skill level of children at the time of the intervention. In addition, we only looked at studies conducted between the years of 1995 and 2020, which had been published either in English or Amharic.

The main quantitative studies included use either experimental or quasi‐experimental designs. To maximize external validity and address second‐order research questions, we also included non‐experimental quantitative studies (e.g., longitudinal or cross‐sectional regression analysis) in our systematic review. However, we excluded these latter studies from our meta‐analysis because of concerns about the biasedness of their impact estimates. For qualitative studies, all studies examining an intervention were included in this review regardless of the methodology.

Lastly, for quantitative studies, we only considered those that looked at literacy outcomes. We included studies that assessed the impact of LOI choices in any or all the following types of skills: emergent literacy sub‐skills, oral language skills, metalinguistic awareness, sound‐symbol correspondences, decoding, oral reading fluency, and reading comprehension. Conversely, outcome measures were not considered as a filter for qualitative studies, which we used to address the secondary research question.

### 2.4 Search methods

Our search strategy enabled us to identify relevant published and unpublished literature by focusing on academic and institutional databases, citation tracking, and snowballing of pertinent references. We performed comprehensive electronic searches across 14 paid‐access and 8 open‐access databases. In addition, we thoroughly examined over 14 key institutional websites and performed generic searches via Google and Google scholar with the aim of identifying relevant gray literature.

We developed search strings in collaboration with an information specialist with the aim of conducting a comprehensive and systematic search across databases. The search strings were developed to return studies that include at least one keyword in the following three themes:

1. Participants: preschool, elementary school, pre‐primary, kindergarten, primary school, early childhood.
2. Literacy: reading, literacy, MT, LOI, medium of instruction, cross‐language transfer, language transition, reading transfer, multilingual education, bilingual education.
3. Setting: low‐income, middle‐income, third world, developing, underdeveloped, LMIC, global south, Africa, Asia, LAC, Southeast Asia.

We assessed the inclusion criteria for study design, comparison condition, and outcomes during the screening of the studies.

### 2.5 Data collection and analysis

After our initial search was complete, we conducted a manual abstract review process. Each abstract was reviewed independently by two trained reviewers and results of this initial review were collated to select studies for the second screening. In the second phase, we reviewed the full text of all studies that passed Phase 1 screening. Again, each full text was reviewed independently by two trained reviewers, this time using specific quantitative and qualitative screening protocols. We compared reviewer scores and again moved forward only those studies deemed worthy of inclusion in our final set.

Our final included studies were then reviewed for quality (qualitative) and risk of bias (quantitative) using rigorous tools. We extracted information from each quantitative study to estimate the standardized effect sizes as Hedge's *g* across studies. We also calculated standard errors and confidence intervals, where feasible. We then pooled the results of the quantitative studies that focused on the same outcome variables and interventions using meta‐analysis in Stata.

For the meta‐analysis, we included only studies with an emphasis on LOI choice that use one of the following designs: (1) experimental designs using random assignment to the intervention or (2) quasi‐experimental designs with non‐random assignment (such as regression discontinuity designs, “natural experiments,” and studies in which participants self‐select into the program). We used random‐effects meta‐analysis because the average effect of LOI choice is likely to differ across contexts due to differences in program design and contextual characteristics.

For the quantitative studies which were not appropriate for meta‐analysis (i.e., non‐experimental studies and those in a category for which there were not enough studies to conduct a meta‐analysis), we produced a narrative synthesis of the studies. This synthesis aimed to identify key learnings from the literature on the effectiveness of LOI policies on literacy outcomes stemming from quantitative studies.

For qualitative studies, we used a “best‐fit” framework synthesis approach to synthesize the findings from the studies to further understand how the hypothesized factors in our theory of change explain the influence of LOI practices and policies on literacy outcomes (Carroll et al., 2013). The main goal was to understand how the qualitative studies explain or contradict the theory of change and quantitative evidence.

Lastly, we conducted an integrated synthesis drawing on the findings of the meta‐analysis, the quantitative narrative synthesis, as well the qualitative synthesis to provide a cohesive picture of the impact of LOI programs on literacy outcomes, the intermediary outcomes of quality of teaching and learning materials, teacher quality, student motivation, and parental and community engagement in literacy learning.

### 2.6 Results

Overall, we identified 45 relevant studies to include in our final review and analysis. In our meta‐analysis, we find positive effects of MT interventions—especially high‐quality and more expensive programs—on letter knowledge (ES = 0.28 SMD, 95% CI = 0.03, 0.52), sentence reading (effect size [ES] = 0.19, standardized mean difference [SMD], 95% confidence interval [CI] = 0.04, 0.34), reading comprehension (ES = 0.29 SMD, 95% CI = 0.12, 0.45), and writing (ES = 0.69 SMD, 95% CI = 0.56, 0.81) in the students’ MT. The meta‐analysis also shows positive effects on reading comprehension (ES = 0.32 SMD, 95% CI = 0.02, 0.63) in the national language. A narrative quantitative synthesis finds positive effects on word reading, reading comprehension, and writing in the later acquired language. A meta‐analysis further finds null effects of word reading in the MT (ES = 0.02 SMD, 95% CI = −0.04, 0.09). However, our results suggest publication bias in studies that examine the effect of LOI on literacy outcomes regardless of the language or outcome measured.

Results from our qualitative meta‐narrative suggest the main activities necessary for a successful LOI program are the presence of high‐quality teaching and learning materials in the MT. Moreover, primary outputs of the MT studies seem to have been related to improved curriculum including display and use of bilingual materials in classrooms, and a positive reception and wide support of MT‐specific learning materials such as textbooks by students and teachers alike. Intermediate outcomes of Mother Tongue Education (MTE) included positive perceptions of results related to teaching quality and increased student motivation while final outcomes from MTE studies include perceived improvements in first language and second language reading skills, especially in comparison to students who were not studying in the MTE schools. English medium programs may have led to numerous perceived positive outcomes. One such outcome was evidence that “language supportive learning” supports learners’ reading and oral skills in second or later acquired languages. Furthermore, there was a reported increase in learners’ confidence when speaking English. Perceived negative outcomes of the English medium programs included a loss in reading and writing skills in the learners’ MT or first language. Additionally, some students reported failing their English courses and/or the content they were being taught in English.

### 2.7 Implications for policy, practice, and research

The findings suggest that high‐quality MT interventions are likely to lead to gains in MT reading outcomes. However, there is a large evidence gap in determining when to transition to a new LOI after the MT.

## 3 BACKGROUND

### 3.1 Description of the problem

Even though more children are in school than ever before, more than 250 million of them are not learning basic literacy and numeracy skills (World Bank, 2018, 2019). Several factors contribute to this state of learning poverty, ranging from the macro (national education and teacher education systems) to the micro (cognitive learning mechanisms). However, one factor central to learning, but often under researched, misunderstood, or overlooked, is the role of language in education. All learning happens in and through language. The language the child is taught in is closely linked to successfully acquiring academic and socioemotional skills.

Children across LMICs are learning in multilingual contexts. This has wide‐ranging social, economic, political, and educational consequences for learning. Yet, LOI programmatic and policy choices are constantly shifting based on forces such as community demands, political realities, and donor policies. Indeed, regions with the highest rates of learning poverty are regions with large mismatches between language(s) children speak and language(s) they are learning and taught in school (World Bank, 2021).

The research evidence is clear that children will learn to read only language(s) they understand (Hoover & Tunmer, 2020; NICHD Early Child Care Research Network, 2005; Ouellette, 2006). Even if children learn to decode words—even with some degree of fluency—reading comprehension will remain unattainable without sufficient oral language comprehension. A few recent systematic reviews highlight the importance of instruction in the MT (or a language the child speaks and understands well) for quality learning outcomes in LMICs (Evans & Acosta, 2020; Nag, Vagh, Dulay, & Snowling, 2019). The benefits to MT‐based multilingual education programs are multifaceted, including higher likelihood of girls and marginalized communities staying in school (Benson, 2005), increasing educational equity and maintenance of cultural and linguistic diversity (Ball, 2010), allowing parents and communities to participate in the learning process (Nag, Vagh, Dulay, & Snowling, 2019) as well as long‐term cost benefits (Heugh, 2004, 2012). There are also clear cognitive benefits to learning to read in a known or familiar language, as the skills from the first language transfer and facilitate learning to read in a new language (Chung et al., 2019; Koda, 2008). Furthermore, evidence indicates that strong bilingual education models have significant positive effects on non‐linguistic functions (Bialystok, 2005) and executive function skills (Bialystok, 2018) that lay a strong foundation for later socioemotional skills, as well as on academic achievement (Collier & Thomas, 2017).

At the same time, there is an ever‐increasing demand from communities for education in the national or international postcolonial language (Coleman, 2011). The primary reason for this demand is the link between the postcolonial language and socioeconomic mobility (Azam, Chin, & Prakash, 2010). Other factors that complicate LOI choices include linguistically heterogenous classrooms, in which there are multiple MTs in one school or area (Nakamura et al., 2017; Reddy, 2011); as well as the fact that some MTs have no scripts, lack teaching and learning materials, have limited trained teachers, or lack political or community will to be implemented as languages in education (Piper, Zuilkowski, & Ong'ele, 2016; Trudell & Piper, 2013).

This leads to a situation in which decision‐makers must reconcile *both* the well‐documented benefits of MT instruction along with the quest for socioeconomic mobility through a postcolonial or international (later acquired) language at earlier grades. Therefore, this systematic review will focus on LOI *transition* choices in education programs and policies on student literacy outcomes in multilingual contexts in LMICs. In particular, we seek to confirm whether MT (or familiar language) instruction impacts reading outcomes, as well as aim to investigate the unanswered question of when to introduce or transition to additional LOI to foster quality bilingual or multilingual reading outcomes.

### 3.2 Description of the intervention

Describing an LOI intervention is complicated as almost all learning takes place through language. All classes, including those in mathematics, science, social studies, and even music and art are taught in language. Learning to read and reading to learn are primarily language‐based skills. As such, all educational interventions have language as one of its key components or ingredients. Given this complexity, we only include studies in which LOI is an identified variable in the design of the study.

Specifically, we included any of the following LOI intervention types:

- Educational programs that utilized children's MT or a familiar language as the initial medium of instruction through primary grades.
- Educational programs in which children in the primary grades are taught in *two or more* languages at the same time.
- Educational programs in which children in the primary grades are taught in a language they do not speak or understand.
- Educational programs in which children in the primary grades are taught in a MT or in a familiar language initially, and then transition to a new language of learning and instruction during the primary school years.

### 3.3 How the intervention might work

#### 3.3.1 Theory underlying bilingual and multilingual literacy acquisition

To base our study in theory (Brown, 2020), we developed a learning science^1^ framework of the *cognitive mechanisms* that underpin literacy learning in bilingual and multilingual learning contexts. These cognitive mechanisms are at the core of a multifaceted, multidisciplinary theory of change of how we expect LOI policy and program interventions to impact literacy outcomes in LMIC

The Cognitive Foundations of Reading and its Acquisition (CFRA) is a model that lays out the cognitive components required for successful reading in *monolingual learners*—and links those components to curriculum effectiveness and reading teacher knowledge and teaching effectiveness (Hoover & Tunmer, 2020). Studies show that teacher effectiveness for improving reading outcomes is significantly related to their own reading enthusiasm (Applegate & Applegate, 2004) as well as their own knowledge of the cognitive foundations of reading (Binks‐Cantrell et al., 2012).

Learning science theories from various disciplines such as psychology and linguistics reveal that the underlying mechanisms of acquisition of reading skills in *bilingual and multilingual learners* is different from learning to read in monolingual learners in significant and predictable ways. First, learning to read in a second or later acquired language is significantly impacted by transfer of reading skills from a first language.^2^ Next, second or later acquired language learning is also significantly impacted by second or later acquired language oral language skills, which are highly variable in bilingual and multilingual learners compared to monolingual learners. This notion that second or later acquired language reading skills are reliant on a combination of MT reading skills and second or later acquired language oral language skills is encapsulated in the linguistic interdependence hypothesis, the underlying proficiency hypothesis (Cummins, 1979, 1981) and the transfer facilitation model (TFM) of second language reading (Koda, 2005, 2007, 2008).

Chung et al. (2019) provide an updated interactive framework for crosslinguistic transfer in second or later acquired language reading, in which they posit that the relationship between MT and second or later acquired language reading skills is influenced by cognitive, linguistic, and metalinguistic factors that include language specific as well as language neutral constructs, MT‐ second or later acquired language distance, MT‐ second or later acquired language proficiency and complexity. The researchers extended the model to postulate that socio‐cultural factors such as age of beginning acquisition of the second or later acquired language, immigration experience, educational settings, and extent of exposure to the MT and second or later acquired language also impact language transfer.

Indeed, empirical evidence is accumulating for each of these factors. In a meta‐analysis of the cognitive and linguistic sub‐skills in transfer, Melby‐Lervåg and Lervåg (2011) find that phonological awareness and decoding skills present significant correlations across MT—second or later acquired language; but that these relationships are less (or not) present in oral language comprehension and reading comprehension subskills. Reflecting this need to start with a foundation in the MT for successful outcomes in the second or later acquired language, Collier and Thomas (2017) present the results from a 32‐year longitudinal research study on bilingual education in the United States. The results reveal that it takes an average of six years of high‐quality instruction in both the MT as well as English/second or later acquired language, with at least 50% of the instruction being conducted in MT for English learners to perform comparably with their monolingual peers on academic outcomes.

There are increasingly diverse research methods being employed to answer a variety of question related to biliteracy development and LOI transitioning policy and practice questions. For example, studies have recently begun using threshold methodologies to examine “how much” of a particular skill or knowledge is needed to benefit from transfer in biliteracy or bilingualism. In Northern England, De Cat, Gusnanto, and Serratrice (2018) utilize Cox proportional hazard regression models to identify a threshold of “bilingual experience” for early executive functioning skill benefits. In North America in a two‐way Spanish‐English bilingual immersion program, Feinauer et al. (2017) employ discontinuous change‐point regression models to show that the relationship between second or later acquired language oral language skills and second or later acquired language reading development is not linear. Finally, in two LMIC contexts—India and Ethiopia—Nakamura and colleagues used structural break regression analyses to test whether there is an empirically determinable point of “sufficiency” in the MT literacy skills to transfer and foster success in literacy skills in the second or later acquired language (Nakamura et al., 2018, 2019). The results of these latter studies showed that there was a nonlinearity in the relationship between first and second language reading scores in six languages pairs across these two countries, implying that there may be a point at which children are cognitively and linguistically ready for literacy instruction in second or later acquired language as MT reading skills reach a point of sufficient maturity for transfer to take place.

Beyond the cognitive underpinnings of biliteracy acquisition, past research identified certain aspects of the home environment as significant predictors of literacy and biliteracy acquisition. According to the Home Literacy Environment (HLE) model, informal language, and literacy practices at home (i.e., those not directly related to engaging with print at home) are predictive of concept of print and emergent literacy skills; whereas formal language and literacy practices at home, (i.e., those that are explicitly meant to teach children language and literacy skills) are predictive of early decoding development (Sénéchal & Le Fevre, 2002, 2014). Cross‐country reviews also highlight that parental attitude toward reading, number of books at home (indirect HLE factors) and literacy‐linked activities at home (direct HLE factors) have a significant impact on reading outcomes (Park, 2008). Given the vast mismatches between home and school language in LMICs (Nag, Vagh, Dulay, Snowling, 2019), as well as the generally lower rates of adult literacy (and thus parental literacy) in LMICs (Abadzi, 2003), the evidence underscores the additional risk of home literacy and language environments that do not have the resources necessary to support reading development in the language of the school – or any language (Nag, Vagh, Dulay, & Snowling, 2019). However, studies also suggest that there is context‐specificity in the relative importance of various dimensions of the home language and literacy environment on specific reading component outcomes (Friedlander, 2020; Nag, Vagh, Dulay, & Snowling, 2019; Park, 2008).

Classroom and teacher factors such as attendance (of both teachers and students), the lack of a safe learning space (Spier et al., 2019), nutritional inputs (Plaut et al., 2017), and availability of print (through digital media or not) are necessary factors for learning—however, they are not sufficient (Snilstveit et al., 2016). Systematic reviews show that pedagogical inputs (such as structured learning progressions, skill‐based learning, teaching, and learning at the “right” level) and teacher professional development are emerging as the most effective ingredients for translating access and safe learning spaces into quality learning outcomes (Evans & Acosta, 2020; Evans & Popova, 2015). Evans and Acosta (2020) underscore MT instruction programs as one of the most effective elements of pedagogical interventions.

Individual differences, such as age and socioeconomic status also are known to moderate the relationship between teaching inputs and language and literacy outcomes. Although it is clear that language learning abilities decline as individuals get older (Flege et al., 1999), there does not seem to be any conclusive evidence that there is a biologically based point at which a child's (or individual's) ability to learn a new language diminishes at a significantly higher rate than before such a point (Bialystok & Hakuta, 1999; Bialystok & Miller, 1999; Birdsong & Molis, 2001; Hakuta et al., 2003). Neurobiological studies reveal that age of acquisition does not alter the underlying brain structure of bilinguals (Frenck‐Mestre et al., 2005; Friederici et al., 2002). However, more intuitively, differential aspects of language learning (such as phonological and grammatic processing) are more susceptible to age‐associated declines than others, such as semantic processing (Abutalebi et al., 2001; Hernandez et al., 2000; Weber‐Fox & Neville, 1999, 2001). De Cat et al.'s (2009) study showed that socio‐economic status (SES) was a significant correlate of bilingualism's positive impacts; but found that the threshold effect of bilingual proficiency held even after SES was controlled for. In the United States and Canada, several evaluations of bilingual education program's impact on learning outcomes shows that although SES is a significant correlate of educational attainments, when controlled for, bilingual children in bilingual programs outperform bilingual children in monolingual programs (Bialystok, 2018). Researchers in the West stress the critical difference between “bilingual education” (positive, additive connotation) and “education of bilingual children” (negative, subtractive connotation) (Bialystok, 2018). This distinction manifests itself in policies that either embrace bilingualism and multilingualism as a force for global integration versus those that use local language as steppingstones toward a different national or international language, which in turn contributes to motivation to learn and community and family involvement in the education system.

Taken together, these studies help us move toward the development of a middle range theory^3^ on multilingual education and biliteracy acquisition. However, it is still unclear how to construct an effective LOI policy, beyond noting that teaching a child in a language they are familiar with is critical for learning. There is little understanding of the mechanism of transfer of skills from one language to another, the “right” timing or skill level at which a child is most likely to benefit from learning a new language, or how to foster quality bilingual/multilingual outcomes after the initial year(s) of MT instruction. As such, the policy question remains at what grade or point to transition students from one medium of instruction to another, and how to develop LOI decisions in LMICs that are likely to be most impactful in improving literacy and biliteracy scores. It is also unknown whether such an LOI model is likely to be the same across different contexts. It is thus critical to consolidate evidence through a rigorous evidence synthesis.

#### 3.3.2 Intervention logic framework

Based on the problem statement above and the theoretical framework of bilingual/biliteracy learning, it is clear that MT and second or later acquired language skills are significantly related in complex and constantly interacting ways that are important for the development of an intervention logic framework. As such, there are many pathways through which language transition interventions can change literacy skills.

**Figure 1:**
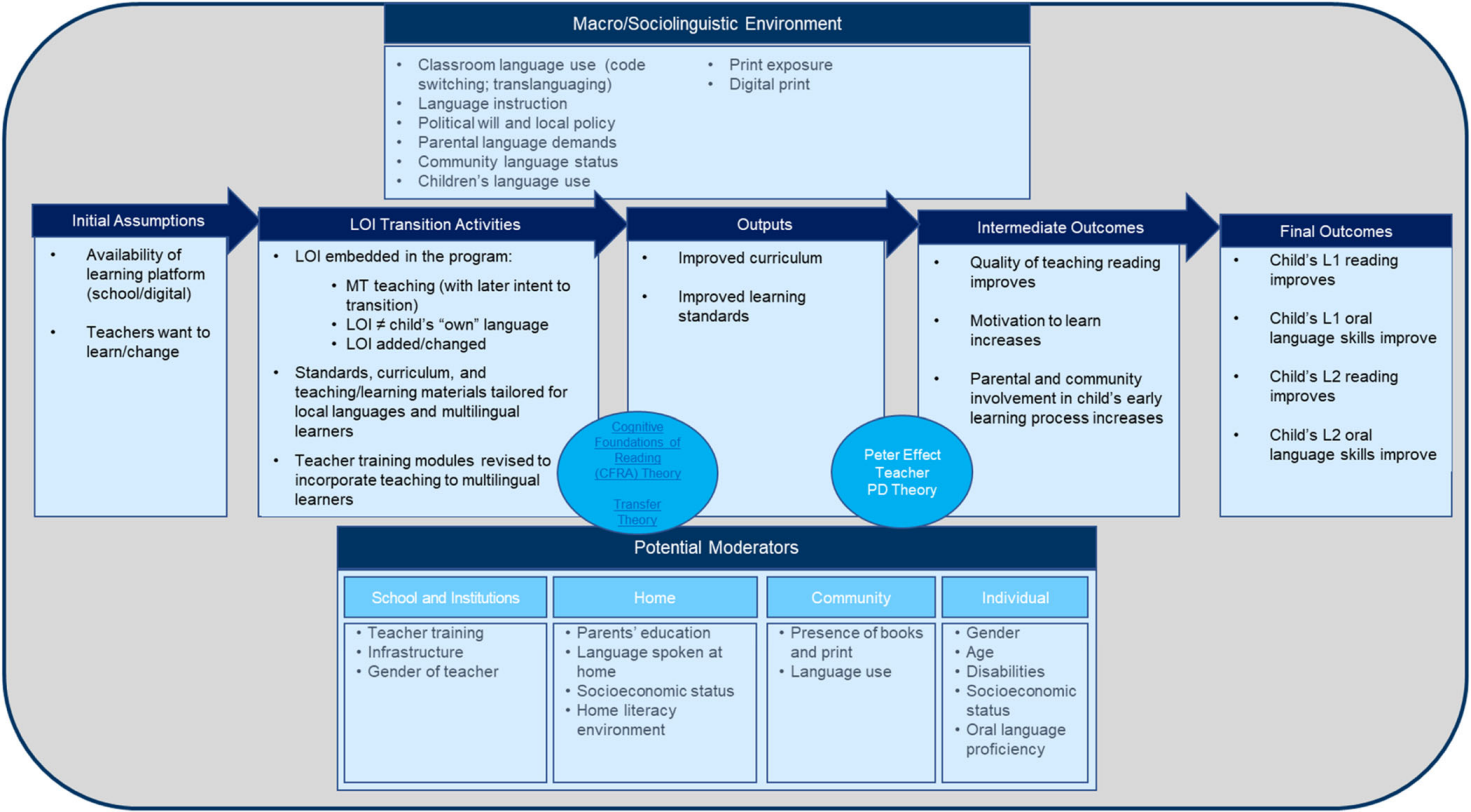
Logic Framework for language of instruction (LOI) policies and interventions on literacy outcomes.

In Figure 1, we present our logic framework. We begin with the key assumption that the child has access to a learning program. Access can occur in the form of school infrastructure with teachers who do not use any technology, or a blended learning environment within a school or community building where teachers or teaching assistants may use some technology to enhance learning (e.g., the eSchool 360 model implemented by the Impact Network in Zambia [De Hoop et al., 2020]), or an online/digital learning program that is—or could be—facilitated by a remote teacher or guide (e.g., Mindspark software), or an entire online/digital learning program that is self‐guided or guided by a virtual built‐in guide (e.g., the Google Bolo app). Another assumption underlying our logic framework is that teachers are willing and able to learn and change how they teach in line with new curricula, teaching materials, and pedagogies tailored to bilingual or multilingual students and varying language types. The introduction of revised LOI choices cannot be effective or adequately applied within the classroom without a revision in the teaching and learning materials to reflect this change. Further, since not all teachers may be fully fluent in the LOI choices or know how to teach those language(s), some may be required to obtain additional trainings to improve teaching knowledge or be re‐assigned to schools where they can teach language(s) they are fluent in and trained to teach reading in.

LOI transition intervention activities can be manifested in many ways. For the purposes of this research, we operationalize LOI transition programs and policies as those that have one or any combination of the following components:

(1) An education program or policy that is implemented in a MT or local language, which will then lead to a transition to (complete change) or addition of (adding as a subject or dual language instruction) teaching of a new language, which may or may not occur during the program. These are important to examine as the skills taught and learned during the program will have significant implications for “readiness” of transfer to the new later acquired language.
(2) An education program or policy that is implemented in a language that is not the child's “own” language (i.e., a language the child has enough proficiency to learn in). These programs are important to investigate as they constitute a child “transitioning” out of their own language into an education system in a new language right from the start of education.
(3) An education program or policy that is implemented in which students transition from one LOI to another or add a new language as part of the medium of instruction during the program or policy.

In any of these scenarios students’ learning acquisition processes can be impacted by the language used for teaching—and as such, the language can have significant implications for effectiveness of learning to read. This is regardless of whether the LOI transition is the key component of the educational program.

Furthermore, the program or policy intervention is most likely to succeed in improving learning outcomes if it has standards, curriculum, and trained teachers (the latter for classroom‐based instruction, as opposed to technology‐based instruction) that focus on the cognitive foundation models (CFRA) (Hoover & Tunmer, 2020) and/or the interactive models of reading transfer (Chung et al., 2019). Although several programs may not explicitly have these theoretical frameworks named in their models, the programs are based on the theoretical premise that a curriculum or a teacher cannot give what they do not have (Applegate & Applegate, 2004; Binks‐Cantrell et al., 2012).

These programmatic components (or activities) may improve the quality of teacher (or technology) knowledge and practices, increase child's motivation to learn (as this will maintain teaching at the “right level,” Banerjee et al., 2016; Pritchett & Beatty, 2015), and increase parental and community involvement in the child's education (Benson, 2005). Finally, all these factors will improve the effectiveness of the LOI decision, leading to impacts on the child's MT literacy skills (reading or decoding based skills as well as oral language skills) and the child's second or later acquired literacy skills.

We also examine the role of several possible factors that are likely to moderate the likelihood that the intervention will improve literacy skills, including community demand for the MT versus the second or later acquired language, local and national policies supporting the implementation of the program or policy, socioeconomic status, parental literacy/schooling level, language(s) spoken at home, home literacy environment (exposure to print), child's initial language use and proficiency level in language(s) of the school, gender, and disability.

Given that LOI choices touch several aspects of the education system, we synthesized the linkages between the inputs to trace how components of LOI programming may impact different sections of the system. For instance, inputs in curricular choices in terms of timing and sequencing of skills in each language would impact standards and curriculum development decisions; whereas teacher training inputs would impact professional development modules, assignment of teachers to schools based on languages they speak versus languages students speak, and urban‐rural teacher availability. All practice and policy recommendations were interpreted within the theory of change, to further develop a middle‐range theory for LOI decision making in LMICs that is reflective of both the micro psycholinguistic and learning science ingredients in improving learning outcomes as well as the macro sociolinguistic, socioeconomic, and political environment within which LOI policy and practice decisions are being made.

### 3.4 Why it is important to do the review

This systematic review aims to help decision makers—ministries of education, teacher training institutes, community leaders, interested donors, and implementing and research organizations—understand and effectively use existing evidence related to multilingual education. Most reading programs funded by large international donors such as USAID and UNICEF include programming in the MT for at least the first few years of primary school (e.g., the USAID‐funded Creative‐implemented Vamos Ler! project in Mozambique or READ II program in Ethiopia, or the USAID‐funded RTI‐implemented PRIMR program in Kenya). Yet, countries continue to shift policies at the national level (e.g., Rwanda switched from a Kinyarwanda LOI policy to an English only policy in 2019 reversing a 2015 MT only policy reform, Edwards, 2019), and regional governments continue to change the years of transition of medium of instruction (e.g., India's regional push toward regional language education despite community demands for earlier English medium of education, Amaravati, 2019; Gejji, 2019). Furthermore, several new programs are rolling out bilingual transition models of reading with limited evidence on the timing of this transition (e.g., USAID Renforcement de la Littearatie au Senegal (Strengthening Literacy in Senegal [RELIS]) Program and the MINEDH Bilingual Transitional Bilingual Model in Mozambique). Indeed, recent international education reports continue to emphasize that “learning poverty” cannot be solved without a better evidence base on how to tackle the role of LOI in multilingual LMIC educational settings (World Bank, 2018, 2019).

This study builds on recent systematic reviews that have shown that MT instruction is critical for learning quality (Evans & Acosta, 2020; Nag, Vagh, Dulay, Snowling, 2019); but is unique in that it will be the first to systematically review the evidence on how and when to add—or transition from—one LOI to another. In other words, it addresses a key policy gap on when to transition from one LOI to a new LOI for improving literacy skills in bilingual or multilingual contexts.

We focused particularly on Ethiopia as the country rolls out its language policy in the Education Roadmap developed in 2020. Our conversations with key stakeholders in the Ethiopian education system, including the Ministry of Education team that developed the roadmap suggest there is an urgent need to gather and understand the evidence on how to implement a multilingual LOI policy effectively. These discussions which took place in 2020 primarily focused on questions related to how much MT instruction is required before introduction of English as a second/later acquired language, as well as when to introduce a third (even later acquired) language.

## 4 OBJECTIVES

To address the evidence gap in making effective LOI decisions, we conducted a systematic review^4^ of the role of LOI choices in education programs and policies on literacy outcomes in multilingual educational contexts in LMICs. Grounded in the multidisciplinary theory of change described above of what factors link LOI choices and literacy outcomes, we gathered, organized, and synthesized the evidence on the specific role of the three LOI choices described in the ToC (teaching in MT with later transition, teaching in a non‐MT language, or teaching in two or more languages at one time) and its impact on literacy and biliteracy outcomes. We focused our systematic review and meta‐analysis on quantitative and qualitative studies of interventions related to the three LOI choices in LMICs as these studies have the highest relevance for decision making in multilingual LMIC contexts. We also only included languages that are relevant and commonly spoken in LMICs. For example, we considered studies that examined Arabic to English transfer, but not Arabic to Swedish transfer. Against this backdrop, we pose the following research questions:

*Primary Research Questions*:

1. What are the short‐ and long‐term impacts of **initial LOI** choices on literacy and biliteracy outcomes, and how do they differ across various LMIC contexts?
2. What are the short‐ and long‐term impacts of **LOI transition** on literacy and biliteracy outcomes, and how do they differ across various LMIC contexts?
3. What qualitative factors support effective implementation of LOI transition policies and practices?

*Secondary Research Question*:

4. What are the evidence gaps about the role of LOI choices in bilingual and multilingual educational contexts in LMICs?

## 5 METHODS

### 5.1 Criteria for considering studies for this review

#### 5.1.1 Types of participants

We included studies that had interventions for primary and secondary school aged children in LMICs, as defined by the World Bank. We included studies about the effects of LOI choices regardless of the educational status or skill level of children at the time of the intervention. Only studies conducted between the years of 1995 and 2020 and published in English or Amharic were included.

#### 5.1.2 Types of studies

The primary research questions on the effectiveness of interventions were addressed using quantitative experimental or quasi‐experimental as well as qualitative studies that include a programmatic or policy intervention.

Specifically, we included the following study designs for **quantitative studies**: (1) experimental designs using random assignment to the intervention and (2) quasi‐experimental designs with non‐random assignment (such as regression discontinuity designs, “natural experiments,” and studies in which participants self‐selected into the program). Quasi‐experimental studies were required to (1) collect longitudinal data (baseline and end line) or cross‐sectional data (end line) from treatment and comparison groups and (2) use propensity score or another type of matching, difference‐in‐differences estimation, instrumental variables regression, multivariate cross‐sectional regression analysis, or other forms of multivariate analysis (such as the Heckman selection model or multivariate ordinary least squares regression analysis). We included studies with data collected at the individual level to ensure that the study focused on child‐level learning outcomes.

We included each of the multivariate quasi‐experimental methods to maximize the external validity of the systematic review. However, several of the quasi‐experimental studies we found included only OLS regression analysis and, therefore, were not able to provide unbiased impact estimates. In such cases, we excluded these studies from our meta‐analysis. Further, we included non‐experimental longitudinal or cross‐sectional studies assessing changes in literacy outcomes over time. These studies were also excluded from our meta‐analysis due to the high level of bias in their outcome estimates. To mitigate concerns about internal validity of some of the included studies, we conducted a risk of bias assessment and stratified our meta‐analysis by identification strategy, where feasible, as in Brody et al. (2015).

At the abstract screening stage, we included all **qualitative studies** that examined an intervention, regardless of methodology. Given the number of studies that were found at this stage, we selected all qualitative studies that studied an intervention (such as a program or policy) for full‐text review. The types of methodologies utilized in our studies were (1) case studies, (2) focus group discussions; (3) key informant interviews; and (4) observations of classrooms or community language use.

#### 5.1.3 Types of interventions

The interventions included in this review were LOI choices made by educational policies and programs that *directly* aimed to increase children's literacy in bilingual or multilingual LMIC education contexts. These interventions included programs with one or more of the following components:

- Full early learning programs for MT education or bilingual and multilingual children
- Official LOI policy (laws or de facto policy) changes
- Teacher training for MT programs or bilingual or multilingual education programs^5^
- Standards development for MT or bilingual or multilingual education programs
- Technology‐based interventions for MT or bilingual or multilingual education programs
- MT or bilingual teaching and learning materials
- MT or bilingual books
- MT or bilingual book clubs, libraries, community reading spaces, mobile book vans etc.
- Assessments used as part of MT of bilingual programming
- MT or bilingual or multilingual radio or media programming

#### 5.1.4 Types of comparison conditions

Eligible comparison conditions included no intervention, pipeline, or “business as usual.” Where the typical comparison condition of “no intervention” or “business as usual” is not selected, eligible comparison conditions included students before a LOI policy change within a region, students within regions with different or no LOI transition policies within a country, and students in schools who are not in a MT or regional language program. In the case of qualitative studies, a comparison was not necessary.

#### 5.1.5 Types of outcome measures

We included studies that focus on intermediate and/or final outcomes. Below we present definitions for each of the outputs and intermediate and final outcome variables for the primary research questions:

*Outputs*:

1. **Curriculum and standards**: We define the curriculum as a document or framework that spells out a wide range of content, objectives, methodologies, resources, assessments, and organizational information regarding what a child is expected to learn. A curriculum should be closely aligned with content standards (what students should know and be able to do at a particular grade) and performance standards (a scale to indicate how, and what percentage of students are performing on the content standards). These standards in turn are of high quality when they are linked to the cognitive foundations of reading acquisition (Hoover & Tunmer, 2020).

*Intermediate outcomes*:

1. **Reading teacher quality**: Based on the Peter Effect studies that a reading teacher cannot pass on what they themselves do not have, we define two main dimensions in reading teacher quality: (a) the enthusiasm to teach reading as indicated by the habits and practices of the teacher (Applegate & Applegate, 2004) as well as (b) the knowledge of the sub‐constructs required for learning to read (Binks‐Cantrell et al., 2012).
2. **Student motivation**: Defined as a child's motivation to attend school as well as to want to learn to read or engage with language, print, or stories. This is usually measured through student's reading behaviors at home or in school and in the community, and attitudes toward reading.
3. **Parental and community involvement**: Studies have found that there is a significant association between student's being taught in home and community languages and the parental involvement in the education system (Benson, 2007). We define this outcome as the frequency and quality of interactions between the parents and/or community members and teachers, as well as the amount of time spent by teachers involved with student's learning at home (helping with homework, learning *from* the student, supporting the student with their learning, asking questions about school, etc.)

*Final outcomes*:

We provide operational definitions for our final outcomes from a range of research on reading development that looks at reading across language and orthographic types as well as across MT and second or later acquired language learning status (Koda & Zehler, 2008; UNSECO UIS et al., 2019; Verhoeven & Perfetti, 2017). Each of these skills were considered for both language types:

1. **Emergent literacy sub‐skills**: Emergent literacy skills or “concept of print (or print awareness),” refer to the ability to understand the conventions and functions of print in one's own language(s), including the ability to distinguish pictures from letters or words, identify the beginning and ending of stories, and so forth.
2. **Oral language skills**: These skills include a range of receptive and expressive oral language abilities, such as the ability to follow simple spoken instructions, identify the meaning of spoken word, explain the meaning of a word in their own words, retell a short story, understand explicit and implicit comprehension questions from a short listening passage, and so forth.
3. **Metalinguistic awareness**: Metalinguistic awareness is the awareness of the functionally useful units of speech and print and the relationship between the two. There are several sub‐components of metalinguistic awareness, but we focus on **phonological awareness**, or the awareness of speech units in any language and **morphological awareness**, or the awareness of morphological units in any language.
4. **Sound‐symbol correspondences**: Oftentimes called letter naming or letter knowledge, sound‐symbol correspondence skills refer to the ability to see a single printed letter, akshara, or character and be able to sound out the symbol.
5. **Decoding**: The ability to see a printed word or cluster of symbols and sound out the word or cluster of symbols. There are several paths that learners take to acquire this skill, but our primary concern is whether students can reach the entire phonological representation of the printed word, regardless of which path they take to achieve the skill.
6. **Oral reading fluency**: The ability to sound out a short passage or story with accuracy, speed, and prosody.
7. **Reading comprehension**: The ability to comprehend both explicit and implicit information presented in single or multiple phrases or sentences of text.

When data was not available for each of these subskills separately, and based on the CFRA (Hoover & Tunmer, 2020), we created composite scores for the emergent literacy and oral language measures (#1–3 above), and for the decoding scores (#4–6 above), and for the reading comprehension scores (#7). Understanding that there are usually only 4–5 questions on many EGRA scores for reading comprehension, we considered either removing these items or merging them with the decoding scores for reliability, if necessary.

We included only literacy outcomes even if the study looked at scores on other subjects. Outcome measures were not considered to filter qualitative studies, which served to explicitly address the secondary research question.

### 5.2 Search methods for identification of studies

We developed a search strategy in consultation with an information specialist. Our search strategy enabled us to identify relevant published and unpublished literature by focusing on relevant academic and institutional databases, citation tracking, and snowballing of references. We identified the following literature searches.

#### 5.2.1 Electronic searches

Comprehensive database searches included the following paid‐access and free‐access electronic databases:

*Paid‐Access Databases*

1. ERIC (Education Resources Information Center)
2. Education Source
3. EdWorkingPapers
4. EdArXiv
5. Registry of Efficacy and Effectiveness Studies (REES)
6. PsycINFO
7. JSTOR Arts & Sciences I‐X Collections
8. JSTOR Business III Collections
9. SAGE Publications
10. ScienceDirect
11. Springer Science+Business Media
12. Taylor & Francis
13. Wiley
14. WorldCat

*Open‐Access Databases*

15. Campbell Collaboration
16. Cochrane Library
17. 3ie Impact Evaluation Repository
18. Directory of Open Access Journals (DOAJ)
19. Directory of Open Access Books (DOAB)
20. Development Experience Clearinghouse (DEC)
21. Institute of Development Studies (eldis)
22. Education International

*Gray Literature*

Gray literature searches included a review of institutional websites as well as generic searches such as via Google and Google Scholar. In addition to the following institutional websites, the team examined key papers in search of other relevant institutional sources.

*Institutional websites or research funders*

1. The UK Department for International Development (DFID) (https://www.gov.uk/government/organisations/department-for-international-development)
2. The US Agency for International Development (USAID) (https://www.usaid.gov/)
3. The Joint Libraries of the World Bank and International Monetary Fund (JOLIS) (https://library.worldbankimflib.org/)
4. J‐PAL (http://www.povertyactionlab.org)
5. UNESCO (https://en.unesco.org/)
6. UNICEF (https://www.unicef.org/)
7. UNICEF Office of Research (https://www.unicef-irc.org/)
8. UNHCR (https://www.unhcr.org/)
9. Population Council (https://www.popcouncil.org/)
10. World Vision (https://www.worldvision.org/)
11. Save the Children (https://www.savethechildren.org)
12. Plan International (https://plan-international.org/)
13. Organization of American States (OAS) (http://www.oas.org/en/)
14. Global Partnership for Education (GPE) (https://www.globalpartnership.org/)

#### 5.2.2 Other searches

Forward and backward snowballing of the references of key papers provided additional studies for review that may not be found in database searches. The team conducted citation searches in Google Scholar, Scopus, and Web of Science. The set of key papers included:

*Key papers*

1. Anchor key papers identified by the authors. These papers are listed below.
2. Key papers identified by external funder (CEDIL).
3. Studies that passed the inclusion criteria.
4. Additional key papers identified from institutional websites.

Table 1 presents the list of key papers identified by authors and reviewers that were used in citation searches.

**Table 1:** Key papers.

Study
Piper, B., Zuilkowski, S., & Ong'ele, S. (2016). Implementing mother tongue instruction in the real world: Results from a medium‐scale randomized controlled trial in Kenya. *Comparative Education Review*, *60*, 776–807.
Brunette, T., Piper, B., Jordan, R., King, S., & Nabacwa, R. (2019). The impact of mother tongue reading instruction in twelve Ugandan languages and the role of language complexity, socioeconomic factors, and program implementation. *Comparative Education Review*, *63*, 591–612.
Banerji, R. Berry, J., & Shotland, M. (2017). The impact of maternal literacy and participation programs: Evidence from a randomized evaluation in India. *American Economic Journal: Applied Economic*, *9*(4), 303–337.
Chicoine (2019). Schooling with learning: The effect of free primary education and mother tongue instruction reforms in Ethiopia. *Economics of Education Review*, *69*, 94–107.
Laitin et al. (2019). The legacy of colonial language policies and their impact on student learning: Evidence from an experimental program in Cameroon. *Economic Development and Cultural Change*, *68*(1), 239–272.
Shin, J., Sailors, M., McClung, N., Hoffman, J. V., Pearson, D., Kaambankadzanja, D., & Mwale, L. (2019). Access to local books: The effects of Read Malawi from a children's rights perspective.

#### 5.2.3 Search strings

We developed search strings in collaboration with an information specialist. Each search string was adapted to fit the syntax of the database host to utilize Boolean operators (AND/OR), wildcards, truncation, and other database search features. The research team finalized the search strings by March 2020. The search strings were designed to return studies that include at least one keyword in the following three themes:

1. Participants: preschool, elementary school, pre‐primary, kindergarten, primary school, early childhood
2. Literacy: reading, literacy, MT, LOI, medium of instruction, cross‐language transfer, language transition, reading transfer, multilingual education, bilingual education
3. Setting: low‐income, middle‐income, third world, developing, underdeveloped, LMIC, global south, Africa, Asia, LAC, Southeast Asia

To capture both quantitative and qualitative literature relevant to the primary research questions, we did not include search strings for study design, comparison condition, or outcome measures. Using these criteria in the search strategy would have led to the exclusion of relevant qualitative studies as well as quantitative and mixed‐methods studies that omit this information from the title and abstract. We assessed the inclusion criteria for study design, comparison condition, and outcomes during the screening of the studies.

### 5.3 Data collection and analysis

#### 5.3.1 Selection of studies

Screening took place in two phases: first on the basis of titles and abstracts and then on the basis of full texts. The research team completed searches by April 2021.

*Screening Phase 1*

After our initial search, we conducted a manual abstract review process. Two trained reviewers independently reviewed each abstract. We conducted the following steps in the abstract screening phase (based on Polanin et al., 2017):

1. Creation of the abstract screening tool. The team created the tool taking into consideration whether:
  a. Questions were objective and “single‐barrelled”
  b. Questions included yes/no/don't know structures, with dropdown options as needed
  c. Detailed key for dropdown options (such as what is meant by “literacy outcome” or “school‐age”)
  d. Questions were ordered hierarchically to ensure that studies that are not meeting any one inclusion criteria are immediately removed from further screening
2. Review of the tool by the lead of our quantitative analysis
3. Training reviewers on the use of the tool
4. Conducting pilot tests of using the screener with the review team all screening the same 15–20 abstracts until consensus is reached on the tool.
5. Periodic check‐ins to enhance intellectual buy‐in as well as to ensure any disagreements are resolved

*Screening Phase 2*

In the second phase, we reviewed the full text of all studies that passed Phase 1 screening. Multiple reviewers independently identified and confirmed the following information for each study:

*Quantitative studies*

- Target population
- Intervention type
- Comparison group
- Quantitative methodology
- Data source
- Outcome measures

*Qualitative studies*

- Target population
- Intervention type
- Qualitative methodology
- Barriers to intervention effectiveness
- Facilitators of intervention effectiveness

Studies passed Phase 2 screening if the information pulled from each study passed the inclusion criteria listed in the previous section. We resolved disagreements through discussion.

#### 5.3.2 Data extraction and management

Two team members with expertise in quantitative research worked independently to extract information from each quantitative study included in the review. Both team members used a data extraction form to compile relevant information for calculating effect sizes.

#### 5.3.3 Assessment of risk of bias in included studies

##### Quantitative

Quantitative studies varied in their degree of rigor and quality; therefore, it was imperative to gauge the quality of the included studies as they were not all equivalent. We assessed each included studies’ risk of bias as an estimate of its quality and rigor. To assess the risk of bias of quantitative studies, we used a tool consisting of 71 criteria related to selection bias, performance bias, outcome and analysis bias, and other biases (Hombrados & Waddington, 2012). Within this tool we extracted information from the studies to determine whether there was low, medium, or high risk of each type of bias. The full quantitative risk assessment tool along with full results of the risk assessment are included in the Additional Figures and Tables section of this report (see Supporting Information: Figure A1).

While the risk of bias assessment was very labor intensive, the number of quantitative studies which required this assessment was low. We assessed the risk of the following biases:

1. Selection bias and confounding, based on quality of identification strategy to determine causal effects and assessment of equivalence across the beneficiaries and nonbeneficiaries.
2. Performance bias, based on the extent of spillovers to students in comparison groups and contamination of the control or comparison group.
3. Outcome and analysis reporting biases, including:
  a. The use of potentially endogenous control variables
  b. Failure to report nonsignificant results
  c. Other unusual methods of analysis
4. Other biases, including:
  a. Motivation and courtesy biases (Hawthorn effect and John Henry effect)
  b. Coherence of results
  c. Retrospective baseline data collection
  d. Differential attrition bias

We also assessed other biases, such as strong researcher involvement in the implementation of the intervention.

##### Qualitative

We assessed the quality of the qualitative studies using the nine‐item Critical Appraisal Skills Programme Qualitative Research Checklist (Critical Appraisal Skills Programme, 2013), judging the adequacy of stated aims, the data collection methods, the analysis, the ethical considerations, and the conclusions drawn. For each item, trained researchers on our team independently filled out the appraisal to determine whether the study had adequately met the item and gave “yes,” no,” or “can't tell” response. Afterwards, the researchers came together to discuss their responses to each item until they reached consensus. We rated studies that scored 0–2 “no” or “can't tell” responses as low risk of bias, studies that scored 3–5 “no” or “can't tell” responses as medium risk of bias, and studies that scored 6–9 “no” or “can't tell” responses as high risk of bias.

After full‐text review, we conducted a thematic synthesis of the qualitative study findings. Each study's main findings were coded to encapsulate the content of each finding (e.g., “the teacher joins Portuguese and the local language to help the student understand,” “we use [the local language] only to pull the student from where he is and understand the subject”). These statements were then categorized into higher order themes (such as “use of local language in post‐colonial language classes”). We then extracted implications for better understanding why or how multilingual education choices worked in various contexts.

Additionally, to further determine the quality of the relevant qualitative studies, the research team used a Qualitative Evidence Review (QER) protocol developed by AIR researchers. See Supporting Information: Figure A2 for the Evidence Review Protocol in the Additional Figures and Tables section of this report. The QER protocol includes eight quality criteria with a total of 28 review questions against which reviewers rated sections of the full text qualitative studies as high, medium, low, or unclear. The QER protocol's criteria topics covered research statement, methodology, research design, recruitment and sampling strategies, appropriate data collection methods, strength of data analysis, clarity of findings and adequate discussion, and value of research.

Reviewers marked an “x” under only one category of quality for each (High or Medium or Low or Not Applicable or Not mentioned) review question. An “x” was counted as “1” point, with the maximum number 28 points. The QER protocol's evidence quality categories are provided as below:

(a) High: Level of evidence is strong
(b) Medium: Level of evidence provided is adequate but not sufficient
(c) Low: Level of evidence provided is weak
(d) Not Applicable: The criteria are not applicable to this research
(e) Not Mentioned: No evidence is provided for the criteria

Reviewers rigorously scanned the relevant full text studies to determine quality of themes provided in the study, collected the points under the high evidence quality category and further screened studies which collected at least 18 points as high‐quality studies. This step resulted in eight high quality studies with qualitative methodologies. See Supporting Information: Figure A3 in the Additional Figures and Tables section of this report for the scoring sheet.

#### 5.3.4 Measures of treatment effect

In accordance with, Chinen et al. (2017), we extracted information from each quantitative study to estimate the standardized effect sizes (for continuous variables) across studies. We also calculated standard errors and confidence intervals where feasible.

We report effect sizes as Hedge's g and adjust effect sizes reported as Cohen's d to Hedge's *g*. We use Hedges’ *g* effect sizes (sample‐size‐corrected SMDs) for continuous outcome variables, which measure the effect size in units of standard deviation of the outcome variable.

The SMD using Cohen's *d* is calculated by dividing the mean difference with the pooled standard deviation by applying the formula in the following equation:

(1) $$\mathrm{SMD}=\frac{Y_{t}-Y_{c}}{S_{p}},$$

where *Y*_t_ refers to the outcome for the treatment group, *Y*_c_ refers to the outcome for the comparison group, and *S*_p_ refers to the pooled standard deviation.

The pooled standard deviation S_p_ was be calculated by applying either Equations (2) or (3):

(2) $$S_{p}=\frac{\sqrt{\left(\left({{\mathrm{SD}}_{y}}^{2}\right)\times \left(n_{t}+n_{c}-2\right)\right)-\left(\frac{\beta^{2}\times (n_{t}\times n_{c})}{n_{t}+n_{c}}\right)}}{n_{t}+n_{c}},$$

(3) $$S_{p}=\frac{\sqrt{\left(n_{t}-1\right)\times s_{t}^{2}+\left(n_{c}-1\right)\times {s_{c}}^{2}}}{n_{t}+n_{c}-2},$$

where SD_*y*_ refers to the standard deviation for the point estimate from the regression, *n*_t_ refers to the sample size for the treatment group, *n*_c_ refers to the sample size for the comparison group, and *β* refers to the point estimate. We used Equation (2) for regression studies with a continuous dependent variable and Equation (3) when the study provides information about the standard deviation for the treatment group and the comparison group.

To transform Cohen's *d* into Hedge's *g*, we used the small sample correction for the SMD by applying on the following formula:

(4) $${\text{SMD}}_{\text{corrected}}={\text{SMD}}_{\text{uncorrected}}\times \left(1–\frac{3}{4\times \left(n_{t}+n_{c}-2\right)-1}\right).$$

Lastly, Equation (5) estimates the standard error of the SMD:

(5) $$=\sqrt{\frac{n_{t}+n_{c}}{n_{c}\times n_{t}}+\frac{{\mathrm{SMD}}^{2}}{2\times (n_{c}+n_{t})}}.$$

##### Methods for handling dependent effect sizes

Where studies report more than one effect size on the basis of different statistical methods, we followed the procedure as laid out in Chinen et al. (2017) and selected the effect size with the lowest risk of bias. When studies presented multiple impact estimates for different variables measuring the same construct, we used a sample‐size weighted average to measure a “synthetic effect size.” In cases where more than one study used the same data set (e.g., national level EGRA data) to measure a literacy outcome for the same intervention, we used the effect size from the study with the lowest risk of bias. If the risk of bias was the same, we estimated an average effect size through inverse‐weighted random effect meta‐analysis. In cases where one study measured the same outcome at different points in time, we extracted the effect size by relying on the outcome measure that was measured closest to the time of the measurement in other studies included in the same meta‐analysis. In cases where studies included more than one treatment arm, we included the effect size from the treatment arm with the lowest risk of bias. If the risk of bias was the same, we used the effect size from the treatment arm that is most like the other programs included in the meta‐analysis.

#### 5.3.5 Dealing with missing data

In cases where it was not feasible to estimate the effect size because of missing data, we contacted the researchers to request the missing information to calculate the effect sizes. However, authors did not respond or were not able to provide sufficient information to calculate the effect size, so we did not include those studies in the meta‐analysis. Even so, the studies and their findings are still discussed within our narrative write‐up.

#### 5.3.6 Quantitative synthesis

We pooled the results of the quantitative studies that focus on the same outcome variables using meta‐analysis. In other words, we conducted multiple different meta‐analyses based on outcome variable (the text above describes which reading outcomes were pooled). We examined the heterogeneity of the effect sizes for each outcome across studies and used meta‐regression to model the variation in effect size and used forest plot visualization (Borenstein et al., 2009). We used Stata to conduct the meta‐analyses.

For the meta‐analysis, we included only studies with an emphasis on LOI choice that used one of the following designs: (1) experimental designs using random assignment to the intervention or (2) quasi‐experimental designs with nonrandom assignment (such as regression discontinuity designs, “natural experiments,” and studies in which participants self‐select into the program). We used random‐effects meta‐analysis because the average effect of LOI choice is likely to differ across contexts due to differences in program design and contextual characteristics.

For those studies deemed unfit for meta‐analysis (i.e., those in a category for which there were not enough studies to conduct a meta‐analysis, and included non‐experimental studies), we produced a narrative synthesis of the studies. This synthesis aimed to identify key learnings from the literature on the effectiveness of LOI policies on literacy outcomes stemming from quantitative studies. Members of the research team reviewed the relevant studies and generated a brief summary of the evidence from these papers related to the effect of LOI policies on students’ reading outcomes.

#### 5.3.7 Qualitative synthesis

We used a “best‐fit” framework synthesis approach to synthesize the findings from the qualitative studies to further understand how the hypothesized factors in our theory of change explain the influence of LOI practices and policies on literacy outcomes (Carroll et al., 2013). The main goal was to understand how the qualitative studies explain or contradict the theory of change and quantitative evidence. All relevant studies raised topics around the key components of our defined theory of change (the activity components, outputs, outcomes, and potential moderators and barriers and facilitators).

The research team analyzed qualitative data in two steps: coding, and interpretation of data. We conducted data analysis using NVivo®, a qualitative data analysis software program. We used the components in the ToC to develop a codebook for analysis in NVivo software. For example, each component was assigned a node in NVivo which enabled coders to pull relevant data from studies into the node that corresponds to a component in the ToC. The final codebook template mirrored the ToC and its components. Coders then selected, read, and coded a small, representative sample of papers to finalize the coding structure, ensure interrater reliability, and formulate initial themes to respond to the research question. This coding outline served as the tool for organizing and subsequently analyzing information (Ritchie & Spencer, 1994).

The analysis team then coded the remainder of the high‐quality papers. The coders reviewed each paper, extracted text that corresponded to each ToC component, pasted that verbatim text into the template, and then summarized the key information. Each paper was analyzed to examine the thematic categories that matched from the findings of each paper with each component of the ToC. The team synthesized the characteristics of the high‐quality papers in terms of the interventions, research questions, methodologies applied, and target populations. The research team then reviewed the material for themes related to both the components of the ToC and for themes that did not fit into the components.

#### 5.3.8 Integrated synthesis

After conducting the meta‐analysis, quantitative narrative synthesis and qualitative synthesis, the team developed an integrated synthesis. For this synthesis, we collated the findings of the meta‐analysis, the quantitative narrative synthesis, and the qualitative synthesis to provide a comprehensive overview of the impact of LOI programs on literacy outcomes. We further combined the findings on the impact of LOI programs on the intermediary outcomes of quality of teaching and learning materials, teacher quality, student motivation, and parental and community engagement in literacy learning.

## 6 RESULTS

### 6.1 Description of studies

#### 6.1.1 Results of the search

**Figure 2:**
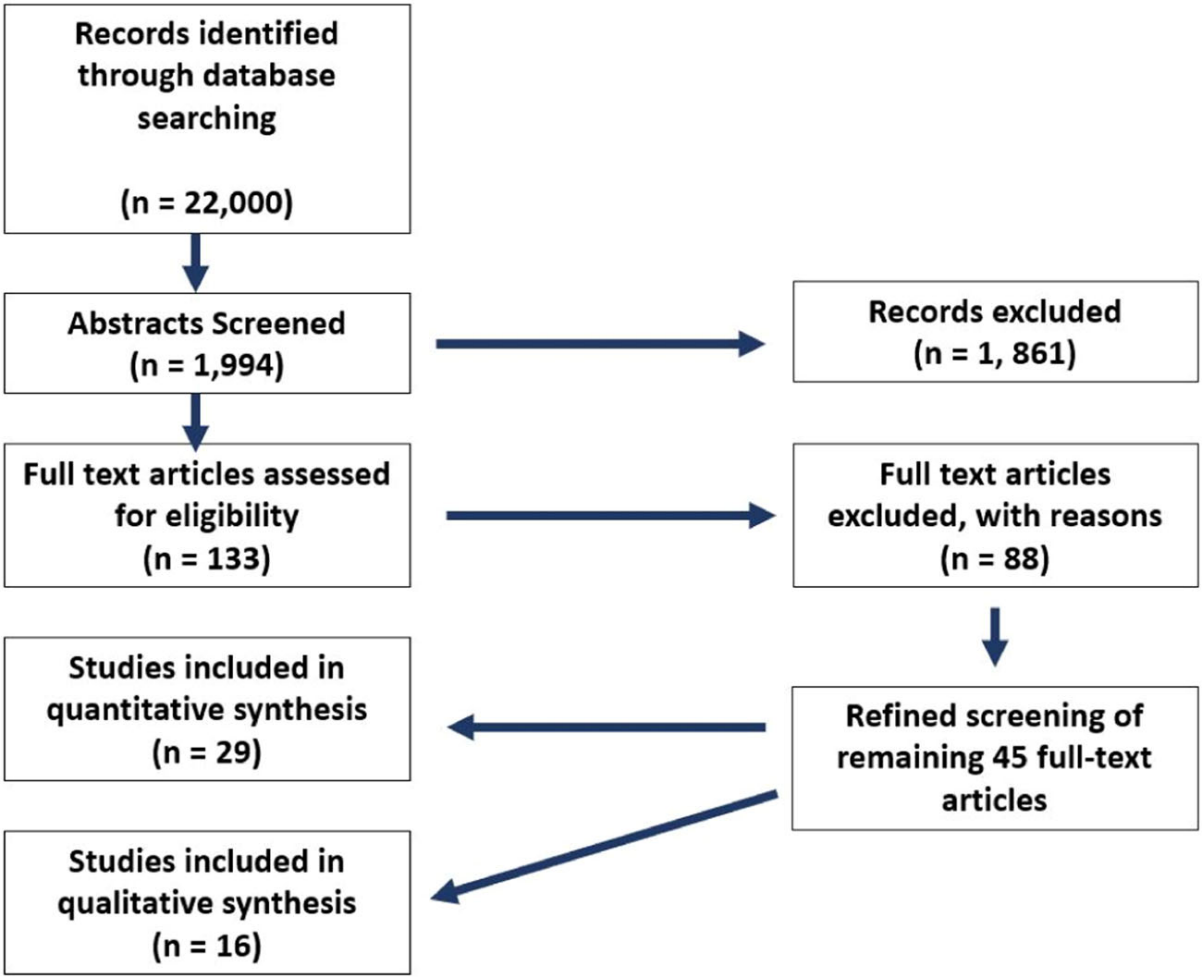
Visual representation of process to identify final included studies.

We identified and screened the titles of over 22,000 records we obtained through database and gray literature searching and selected 1994 of these records for abstract screening. Following a manual double review of these abstracts, 133 records were selected to undergo full text review. The full texts of these references were read in detail by at least two reviewers, and after applying the pre‐determined selection criteria, 82 articles were excluded resulting in 45 studies in the final review. These articles included 29 studies with quantitative methodologies, and 16 studies with qualitative methodologies. Figure 2 illustrates the filtering process.

### 6.2 Excluded studies

Following title and abstract screening, 133 full texts were screened for eligibility. Most of these full texts were excluded (*n* = 88, 62%). We provide information on excluded studies in the reference list. Ultimately, we had 45 studies that were considered relevant for full‐text reviews.

### 6.3 Included quantitative studies

A summary of characteristics of the 29 included quantitative studies can be found in Table 2. As intended in our initial search criteria, all included studies were published in the last 20 years with 93% (*n* = 27) of the studies published in the last 10 years, indicating that rigorous evidence on LOI policies has increased considerably in the last decade.

**Table 2:** List of relevant studies with quantitative methodologies.

	Papers	Countries	Study design
1.	Alptekin et al. (2007)	Turkey	QED
2.	Argaw (2016)	Ethiopia	QED
3.	Awopetu (2016)	Nigeria	Non‐experimental
4.	Brunette et al. (2019)	Uganda	Experimental
5.	Castillo & Wagner (2019)	South Africa	Experimental
6.	Chicoine (2019)	Ethiopia	QED
7.	He et al. (2008)	India	Experimental
8.	He et al. (2009)	India	Experimental
9.	Hynsjo & Damon (2015)	Peru	Non‐experimental
10.	India National Council of Educational Research and Training NCERT (2011)	India	QED
11.	Jain (2013)	India	QED
12.	Jamaludin et al. (2016)	Malaysia	QED
13.	Kerwin & Thornton (2020)	Uganda	Experimental
14.	Lai et al. (2015)	China	Experimental
15.	Laitin et al. (2019)	Cameroon	Non‐experimental
16.	Lee et al. (2015)	Cambodia	Non‐experimental
17.	Lucas et al. (2014)	Kenya; Uganda	Experimental
18.	Milligan et al. (2016)	Rwanda	Non‐experimental
19.	Piper et al. (2016)	Kenya	Experimental
20.	Piper et al. (2018)	Kenya	Experimental
21.	Power et al. (2017)	Bangladesh	QED
22.	Ramachandran (2017)	Ethiopia	QED
23.	Sailors et al. (2010)	South Africa	QED
24.	Santibanez (2016)	Mexico	Non‐experimental
25.	Shin et al. (2019)	Malawi	QED
26.	Simsek & Alisinangolu (2008)	Turkey	QED
27.	Taylor & Coetzee (2013)	South Africa	Non‐experimental
28.	Walter & Dekker (2011)	Philippines	Experimental
29.	Wawire & Kim (2018)	Kenya	Experimental

Abbreviation: QED, quasi‐experimental design.

We included 11 studies with experimental designs (i.e., studies that use random allocation methods), and 11 studies with quasi‐experimental designs (i.e., studies that use non‐random allocation methods such as propensity score matching or eligibility cutoffs to select a comparison group, combined with statistical analysis to address confounding). Finally, we also included seven non‐experimental cross‐sectional and longitudinal studies.

Most of the included studies (55%, *n* = 16) were conducted in sub‐Saharan Africa: Kenya (*n* = 4), Ethiopia (*n* = 3), South Africa (n = 3), Uganda (*n* = 2), Cameroon (*n* = 1), Malawi (*n* = 1), Nigeria (*n* = 1) and Rwanda (*n* = 1). Four studies were performed in East Asia—Cambodia (*n* = 1), China (*n* = 1), Malaysia (n = 1) and the Philippines (*n* = 1)—and 5 were performed in South Asia—India (*n* = 4) and Bangladesh (*n* = 1). Two studies were conducted in Latin America: one in Mexico, and one in Peru. Finally, two studies were conducted in Europe, both of which took place in Turkey.

**Figure 3:**
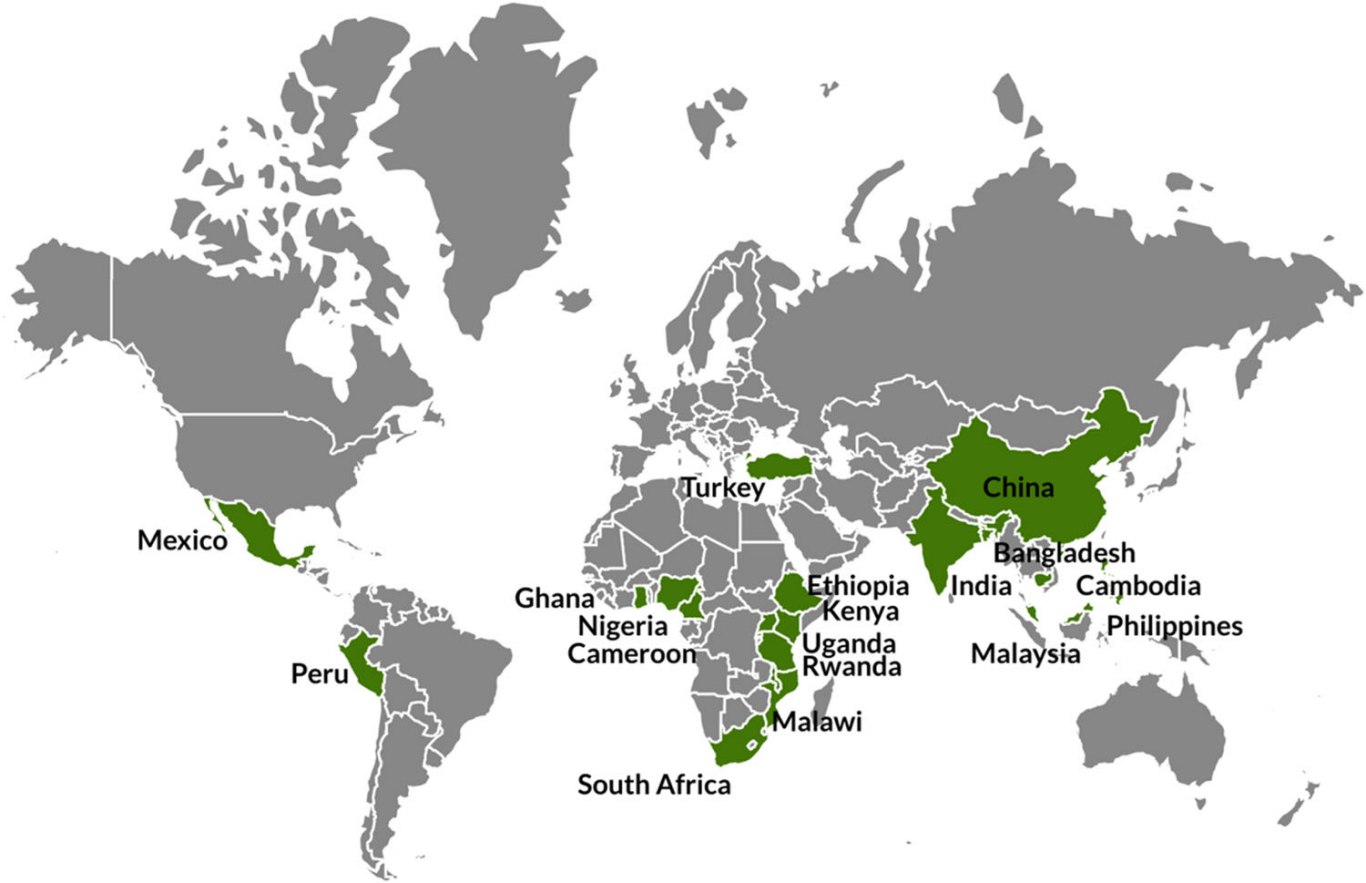
Map indicating the country settings for the studies with quantitative methodologies.

In terms of income levels, most included studies (41%, *n* = 12) were conducted in Lower to Middle‐Income Countries (Bangladesh, Cambodia, India, Kenya, Nigeria, and Philippines). Nine studies were conducted in Upper Middle‐Income Countries (China, Malaysia, Mexico, South Africa, Turkey, and Peru) and 8 studies were set in Lower‐Income Countries (Cameroon, Ethiopia, Malawi, Rwanda, and Uganda). See Figure 3 for a map indicating the country settings of the quantitative studies.

### 6.4 Included qualitative studies

**Figure 4:**
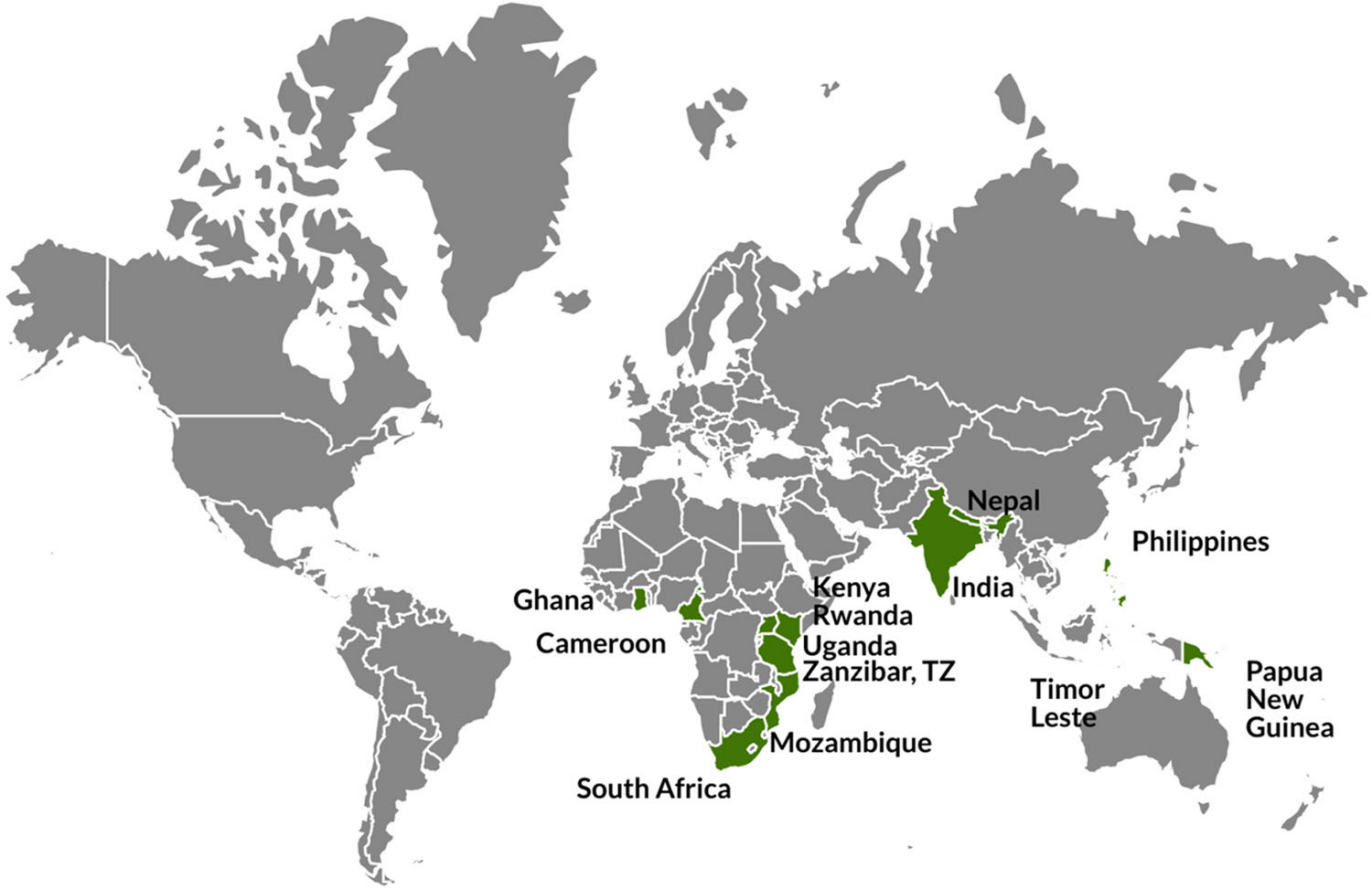
Map indicating the country settings for the studies with qualitative methodologies.

All 16 relevant studies with qualitative methodologies were published in the last 10 years with almost 62% (*n* = 10) of the studies published in the last 5 years. Most included studies (68%, *n* = 11) were conducted in Sub‐Saharan Africa: South Africa, Zanzibar, Cameroon, Kenya, Uganda, Rwanda, and Ghana. Four studies were performed in South Asia and Southeast Asia: Nepal, India, Philippines, and Timor Leste. Finally, one study was completed in the Oceania: Papua New Guinea. Table 3 provides a list of these studies and Figure 4 of the country settings for the qualitative studies.

**Table 3:** List of 16 studies with qualitative methodologies included for full‐text review.

	Papers	Countries
1.	Malebese & Tlali (2019)	South Africa
2.	Caffery et al. (2016)	Timor Leste
3.	Opoku‐Amankwa (2009)	Ghana
4.	Sibomana (2020)	Rwanda
5.	Babaci‐Wilhite (2015)	Zanzibar
6.	Kuchah (2016)	Cameroon
7.	Malone & Paraide (2011)	Papua New Guinea
8.	Mathias & Masagazi Masaazi (2012)	Uganda
9.	Mose (2016)	Kenya
10.	Sah & Li (2018)	Nepal
11.	National Council of Educational Research and Training (NCERT) (2011)	India
12.	Benson (2000)	Mozambique
13.	Benson & Wong (2017)	Cameroon
14.	Brink & Nel (2019)	South Africa
15.	Harden et al. (2020)	Philippines
16.	Milligan et al. (2016)	Rwanda

## 7 RISK OF BIAS IN INCLUDED STUDIES

### 7.1 Risk of bias of quantitative studies

**Figure 5:**
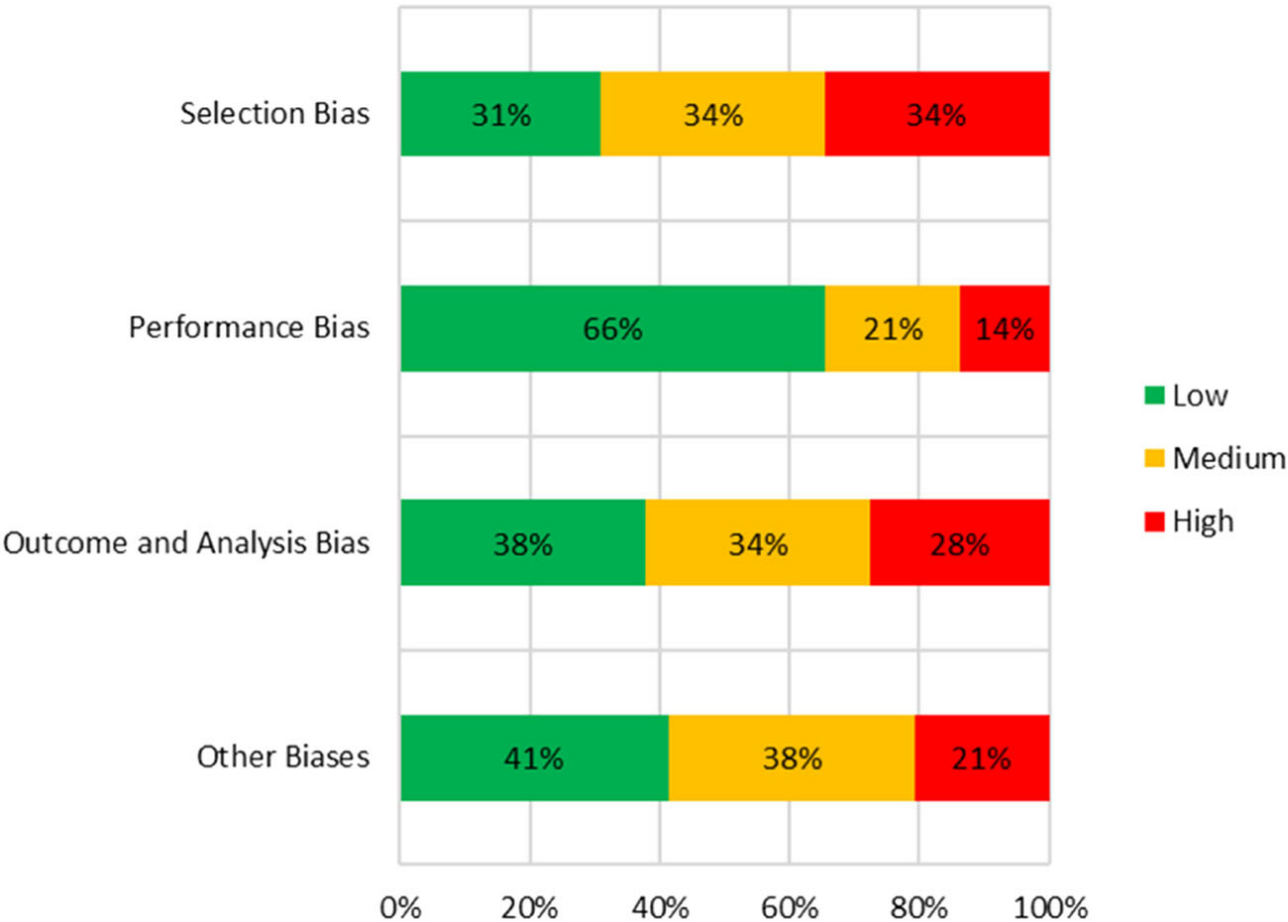
Risk of bias assessment for quantitative studies.

Risk of bias was rated for each study by two individual reviewers. After completing all assessments, the study team compared ratings to determine whether they agreed about all risk ratings. In general, the study team was in alignment on their ratings for all studies. Reviewers disagreed about the risk of selection bias for one study (Taylor & Coetzee, 2013), the risk of performance bias for three studies (Jamaludin et al., 2016; Walter & Dekker, 2011; Wawire & Kim, 2018), the risk of outcome and analysis bias for two studies (Chicoine, 2019; Kerwin & Thornton, 2020), and the risk of other bias for three studies (Kerwin & Thornton, 2020; Lucas et al., 2014; Milligan et al., 2016). In instances of reviewer disagreement, the team went through the studies together and arrived at a consensus. Figure 5 presents the overall results of our risk of bias assessment.

Of the final 29 quantitative studies, nine studies were rated low risk of selection bias. Eight of these studies used random assignment to identify treatment and control groups with sufficiently large sample sizes. The final study used a natural experiment exploiting birth cohorts and the introduction of new education policies. Studies using random assignment to treatment conditions that used small sample sizes or whose control groups were phased into the intervention before the endline data were collected were scored as having a medium risk of selection bias. Further, quasi‐experimental studies using matching techniques with difference‐in‐differences methods to identify comparison groups with undefined characteristics used in the matching process were rated as having a medium risk of selection bias. Similarly, studies which mentioned that schools receiving the intervention differed from those not receiving the intervention in significant ways that were never analyzed were also rated as having a medium risk of selection bias resulting from confounding bias. Finally, studies using nonrandom assignment to treatment conditions while also not examining or correcting for issues related to differing trends or characteristics between treatment and comparison groups were rated as having a high risk of selection bias. Studies where government officials purposively selected schools to receive the intervention or serve as comparison groups with a perceived lack of objectivity or those only using multivariate analyses were also rated as having a high risk of selection bias.

Of the final studies, 19 were rated as having a low risk of performance bias. All these studies evaluated interventions restricted to a particular school separate from the control schools which limited the risk of contamination between the treatment and control group. Further, the interventions were noted as being implemented with fidelity suggesting limited, if any, issues related to program performance. Studies in which treated students and comparison students attended the same schools but were in different classrooms were rated as having a medium risk of performance bias while those in which treated and comparison students were in the same classroom were rated as having a high risk of performance bias.

Eleven included quantitative studies were rated as having a low risk of outcome and analysis bias. These studies measured literacy outcomes using traditional constructs such as scores on tests of student's subskills such as oral language proficiency, word reading, and reading comprehension. Each of these studies measured these skills using a continuous variable and using appropriate methods based on their research design (i.e., difference‐in‐differences used with an RCT). Studies which had a robust research design but only measured literacy skills using a binary literacy variable were rated as having a medium risk of outcome and analysis bias. Studies only including post‐intervention comparisons of treatment and comparison groups were rated as having a high risk of outcome and analysis bias, as were those studies including no baseline measures or including no covariates to disentangle the effect of the intervention from other potential explanatory variables.

**Figure 6:**
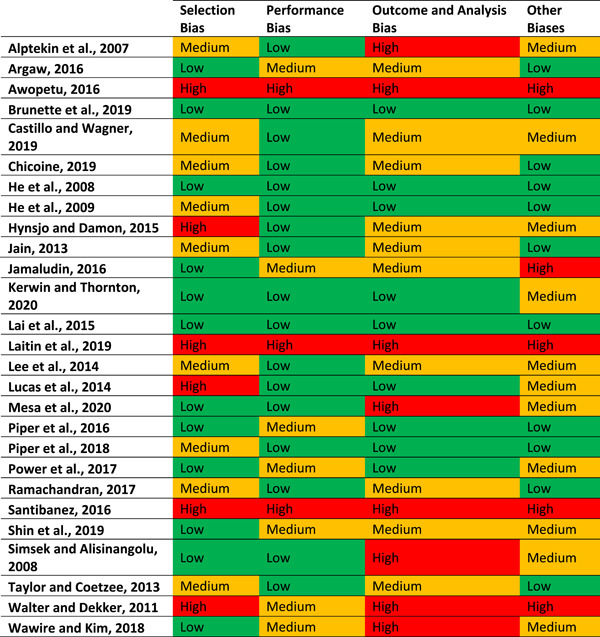
Overview of the risk of bias assessment for included quantitative studies.

Finally, of the 29 included quantitative studies, 12 were rated as having a low risk of other biases. These studies did not exhibit any risk of including other biases. However, the other 17 studies were rated as having medium risk of other biases because they did not account for high rates of attrition. Further, some of these studies failed to correct for preprogram differences in trends between treatment and comparison groups. See Figure 6 for an overview of the risk of bias assessment for included quantitative studies.

### 7.2 Quality of qualitative studies

As described in the Data Collection and Analysis section, to determine the quality of the 16 qualitative studies, the research team used a QER protocol developed by AIR researchers.

After applying the quality appraisal protocol to the sixteen included studies, the eight studies listed in Table 4 emerged as high quality. These eight high‐quality studies were conducted in eight different countries across three regions: Cameroon, Mozambique, Rwanda and Kenya in sub‐Saharan Africa, Timor‐Leste in the pacific region and Nepal, India, and Philippines in Southeast Asia.

**Table 4:** List of eight high quality qualitative studies.

1. Caffery, J., Coronado, G. & Hodge, B. (2016). Multilingual language policy and mother tongue education in Timor‐Leste: a multiscalar approach. *Language Policy*, *15*, 561–580.
2. Kuchah, K. (2016). English‐medium instruction in an English–French bilingual setting: issues of quality and equity in Cameroon. *Comparative Education*, *52*(3), 311–327. ISSN 0305‐0068.
3. Peter Nyakundi Mose. (2016): Bilingualizing linguistically homogeneous classrooms in Kenya: implications on policy, second language learning, and literacy. *International Journal of Bilingual Education and Bilingualism*.
4. Pramod Kumar Sah & Guofang Li. (2018) English Medium Instruction (EMI) as Linguistic Capital in Nepal: Promises and Realities. *International Multilingual Research Journal*, *12*(2), 109–123.
5. Carolyn J. Benson. (2000). The Primary Bilingual Education Experiment in Mozambique, 1993 to 1997. *International Journal of Bilingual Education and Bilingualism*, *3*(3),149–166.
6. Harden, K., Sowa, P., & Punjabi, M. (2020). All Children Reading**—**Philippines. 2019 Language Usage Study in Bahasa Sug, Chavacano, Magindanawn, and Mëranaw Mother Tongue Schools: Findings Report. United States Agency for International Development [USAID].
7. National Council of Educational Research and Training [NCERT]. (2011). Programme Evaluation Report: Multilingual Education in Orissa. SSA‐Technical Cooperation Fund, India.
8. Milligan L. O., Clegg J. & Tikly, L. (2016). Exploring the potential for language supportive learning in English medium instruction: A Rwandan case study. *Comparative Education*, *52*(3), 328–342.

All of the studies had well‐defined research questions or study aims. The mixed method studies that aimed to evaluate programs had explicit qualitative questions such as if the program was being implemented as intended and if there were any impacts of the program. The qualitative studies primarily aimed to understand perceptions regarding the LOI in the schools and motivations behind language instruction. All eight studies analyzed the implementation and concerns of either a bilingual or multilingual LOI policy in a LMIC (with the exception of Nepal as the only country which was not colonized). Three of the above high‐quality studies included English as later acquired language.

## 8 SYNTHESIS OF RESULTS

In this section, we present the results of our various syntheses. First, we discuss the meta‐analysis of the quantitative studies by literacy skill measured. Then, we provide a narrative synthesis of quantitative studies including those we were unable to include in the meta‐analysis. We then present a synthesis of the qualitative studies. Finally, we offer an integrated synthesis in which we draw on the findings from each synthesis.

### 8.1 Synthesis of quantitative studies

This section presents the results of our meta‐analysis of the effects of MT interventions and LOI transitions on students’ literacy outcomes. We present results by literacy skill measured, where feasible, and provide a narrative analysis to determine the separate effects of different types of MT‐based interventions and LOI transitions as well as the differences in effect sizes based on the literacy outcome measured and the language of assessment.

#### 8.1.1 Oral language proficiency

Of the 29 included quantitative studies, three studies focusing on LOI literacy interventions included an impact estimate on oral language proficiency that we were able to include in our meta‐analysis. For example, Piper et al. (2018) ran an RCT to evaluate the USAID‐funded Primary Math and Literacy Initiative (PRIMR) program in Kenya, which introduced innovative teaching methods, new learning materials, and professional development in both English and Kiswahili as well as in local MTs for a subset of schools. Specifically, we examine results from their cohort receiving the MT inputs on students’ oral language proficiency in the national language (Kiswahili). Shin et al. (2019) studied the effects of the USAID‐funded Read Malawi project which developed reading materials and teacher guides in the national language (Chichewa), as well as English. Meanwhile, Lee and co‐authors (2015) study the impact of MT instruction on the national language for ethnic minority children in bilingual schools in Cambodia compared to their counterparts in monolingual schools. We summarize the measurement of the outcome variable and language of measurement in Table 5.

**Table 5:** Measurement of oral language proficiency.

Study	Intervention name	Definition of outcome variable	Grade level	Language
Lee et al. (2015)	Cambodia Bending Bamboo (BB) Bilingual Schools	Oral language	Grade 4	National (Khmer)
Piper et al. (2018)	Kenya Primary Math and Reading (PRIMR) Initiative	Listening comprehension	Grade 1 and 2	National (Kiswahili)
Shin et al. (2019)	Read Malawi	Picture identification	Grade 2 and 3	National (Chichewa); Later Acquired (English)

The table shows that of the few studies that measured oral language proficiency, only one study examined the effect of LOI interventions in MT and national languages on students’ oral language proficiency for a later acquired language. Because of the limited number of studies, we include the estimate from the Shin et al., 2019 study as an example of suggestive evidence of the impact of MT language interventions on oral language proficiency for the later acquired language until other studies assess the same. The estimated effect of LOI interventions in the national and later acquired language on oral language proficiency of a later acquired language (English), as measured through picture identification, is 0.05 standard deviations (SMD = 0.05, 95% CI = −0.06, 0.15), which is not statistically significant.

Further, we were unable to determine the estimated effect size for oral language proficiency from Lee et al. (2015), and while the Piper et al. (2018) and Shin et al. (2019) interventions both focused on including teacher training and materials development, the Piper study focused on producing learning resources in the MT while Shin did so in the national language, indicating that the interventions were too dissimilar to include in one meta‐analysis. Instead, we report the results of these two studies separately. Regarding the impact of LOI interventions in MT on the oral language proficiency of students in their national language, Shin et al. (2019) found an average negative effect (SMD = −0.32, 95% CI = −0.42, −0.23), while Piper et al., 2018 reported null impacts (SMD = −0.00, 95% CI = −0.11, 0.11).

#### 8.1.2 Phonological awareness

We also examined the effects of LOI interventions on students’ phonological awareness. Of the 29 included quantitative studies, only one included estimates of the impact of LOI interventions on students’ phonological awareness. Kerwin and Thornton (2020) evaluate the impact of the full and reduced‐cost versions of the Northern Uganda Literacy Project (NULP), a MT early primary literacy program. The reduced cost version of the intervention removed the most expensive materials, implemented a model in which trainings were delivered by government employees instead of NGO staff, and provided fewer support visits to teachers. In this study, phonological awareness was only examined in the MT rather than in the national or later acquired language. We report the estimated effect size for initial sound recognition in the MT found in Kerwin & Thornton, 2020: 0.65 standard deviations (SMD = 0.65, 95% CI = 0.52, 0.78) for the full program and 0.08 standard deviations (SMD = 0.08, 95% CI = −0.02, 0.17), which was not statistically significant, for the reduced cost intervention (Table 6).

**Table 6:** Measurement of phonological awareness.

Study	Intervention name	Definition of outcome variable	Grade level	Language
Kerwin & Thornton (2020)	Northern Uganda Literacy Project	Initial sound recognition	Grade 1	Mother tongue (Leblango)

#### 8.1.3 Letter and syllable reading

We were able to include four studies that measured the effect of LOI interventions on students’ letter and syllable reading (decoding). Of these, two examined the effect on students’ MT letter knowledge, two on students’ national language letter knowledge, and only one looked at students’ letter knowledge of a later acquired language, specifically English. All studies assessed the effects of the intervention on letter and syllable knowledge for Grade 1 and 2 students. Table 7 provides an overview of the measurement of letter and syllable knowledge by paper.

**Table 7:** Measurement of letter and syllable reading.

Study	Intervention name	Definition of outcome variable	Grade level	Language
Kerwin & Thornton (2020)	Northern Uganda Literacy Project	Letter name knowledge	Grade 1	Mother tongue (Leblango)
Piper et al. (2016)	PRIMR	Letter fluency	Grade 1 and 2	Mother tongue (Lubukusu & Kikamba)
		Syllable fluency		
Piper et al. (2018)	PRIMR	Letter fluency	Grade 1 and 2	National (Kiswahili); Later Acquired (English)
		Syllable fluency		
Shin et al. (2019)	Read Malawi	Letter identification	Grade 2	National (Chichewa)
		Syllable identification		

**Figure 7:**
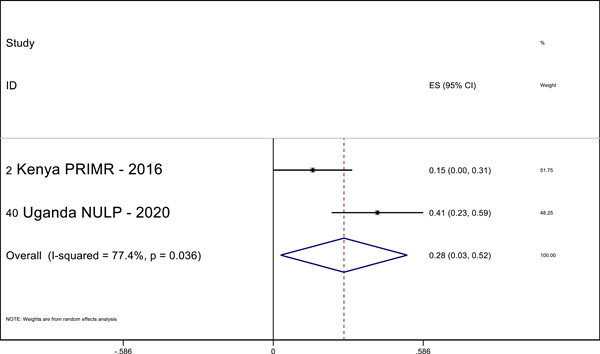
Effects of language of instruction on letter and syllable reading (mother tongue).

For our meta‐analysis, we calculated the average effect sizes for each paper by language type to get an overall effect size by intervention and study. Piper et al. (2016) included the measurement of letter fluency for two different MTs across two different grade levels in their evaluation of a MT‐based reading intervention focused on providing materials and training in local languages. To ensure comparability across studies, these effect sizes were averaged based on sample size weighting down to one average effect size of the program on students’ MT letter knowledge. While we were not able to gain a deep understanding of the impacts of LOI interventions on letter and syllable knowledge as we were only able to include a total of four studies, the results suggest that interventions creating teaching and learning materials in MTs have a positive effect on letter knowledge in the students’ MT and slightly negative effect on letter knowledge for English (the later acquired language) (Kerwin & Thornton, 2020 (reduced); Piper et al., 2016; Piper et al., 2018). Interventions which only focused on teaching and learning material development in the national and later acquired language found a null effect on letter knowledge for the national language (Shin et al., 2019). The average estimated effect of these LOI interventions on students’ MT letter knowledge was estimated as 0.28 standard deviations (SMD = 0.28, 95% CI = 0.03, 0.52; I‐sq = 77%; evidence from Kerwin & Thornton, 2020 and Piper et al., 2016) (Figure 7). The limited number of studies did not allow for any sensitivity or subgroup analyses.

#### 8.1.4 Word Reading

We also synthesized the effects of LOI interventions on students’ word reading by language type. Only five of the 29 quantitative studies included a measurement of word reading assessed as decoding, familiar/known word reading, or invented/nonword reading. While we acknowledge the dual routes of acquiring word reading skills and the fact that sight words could be accessed differently in familiar words versus the graphology‐phonology mapping that is required in invented words (Coltheart, Rastle, Perry, Langdon, & Ziegler, 2001), we collapse these constructs together given their significant overlap (Swanson et al., 2003), especially in more transparent languages. Of these studies, which included the introduction of MT teaching and learning materials (Kerwin & Thornton, 2020; Piper et al., 2016; Piper et al., 2018), the introduction of national and later acquired language teaching and learning materials (Shin et al., 2019), and synthetic phonics instruction to learn a later language (Jamaludin et al., 2016), two examined impact on MT word reading, two examined impact on national language word reading, and two measured word reading in a later acquired language. Table 8 provides the breakdown of measurement, grade level and language by study.

**Table 8:** Measurement of word reading.

Study	Intervention name	Definition of outcome variable	Grade level	Language
Jamaludin et al. (2016)	Synthetic Phonics	Decoding skills	Grade 6	Later acquired (English)
Kerwin & Thornton (2020)	Northern Uganda Literacy Project	Familiar word recognition	Grade 1	Mother tongue (Leblango)
		Invented word recognition		
Piper et al. (2016)	PRIMR	Nonword fluency	Grade 1 and 2	Mother tongue (Lubukusu & Kikamba)
Piper et al. (2018)	PRIMR	Nonword fluency	Grade 1 and 2	National (Kiswahili); Later acquired (English)
Shin et al. (2019)	Read Malawi	Word recognition	Grade 2	National (Chichewa)
		Word reading		

**Figure 8:**
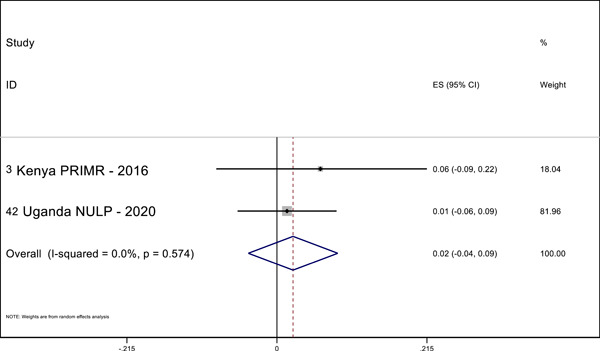
Effects of language of instruction interventions on word reading (mother tongue).

Our meta‐analysis of the effect of LOI interventions on word reading began with the analysis of the effects for MT. As mentioned, only two of our included studies assessed the impact on MT word reading (Kerwin & Thornton, 2020; Piper et al., 2016). The impacts from Kerwin & Thornton, 2020 are those for the reduced cost NULP intervention as this version of the intervention is more comparable with PRIMR. We find the estimated impact is 0.02 standard deviations and not statistically significant (SMD = 0.02, 95% CI = −0.04, 0.09; *I* ^2^ = 0%; estimated from two studies) (Figure 8). While all studies found positive average effects of MT interventions on MT word reading, the confidence intervals of the effects for Piper et al. (2016) and Kerwin and Thornton (2020)'s reduced model all cross zero indicating that the effects are not statistically significant.

For the national language, we present the effects from the PRIMR study and the Read Malawi study separately as these are dissimilar interventions such that we are unable to conduct a meta‐analysis. Piper et al. (2018) found an estimated effect of the MT intervention on Kiswahili word reading of 0.08 standard deviations (SMD = 0.08, 95% CI = −0.01, 0.17) while Shin et al. (2019) found an estimated effect of a Chichewa intervention on Chichewa word reading of 0.001 standard deviations (SMD = 0.001, 95% CI = −0.09, 0.11), both not statistically significant.

Similarly, for the later acquired language, the Synthetic Phonics program and PRIMR examine very different interventions so we could not include these in a meta‐analysis. Jamaludin et al. (2016) investigates the effectiveness of synthetic phonics in the development of early reading skills among struggling young English as a second language (ESL) readers in a rural school in Malaysia. The author's findings suggest that synthetic phonics in ESL could be effective in developing early reading skills in English for low‐achieving ESL readers in grades 1–3. More specifically, Jamaludin et al. (2016) found a significant effect of 4.25 standard deviations (SMD = 4.25, 95% CI = 3.55, 4.95) while Piper et al. (2018) also found a significant effect but of only 0.15 standard deviations (SMD = 0.15, 95% CI = 0.06, 0.24). Of importance, Jamaludin's study boasts a high risk of bias due to the extremely small sample (*n* = 80) used, and the lack of clarity around how students were placed into treatment conditions.

#### 8.1.5 Sentence Reading

Of the 29 quantitative studies, six include sentence reading as a key outcome in their analysis. The majority (four) examine sentence reading in the MT while two additional studies examine sentence reading in both the national language and later acquired language. All six of these studies focus on effects for students in Grades 1–4 (early primary school). Table 9 provides more details of these studies including the measure used.

**Table 9:** Measurement of sentence reading.

Study	Intervention name	Definition of outcome variable	Grade level	Language
Brunette et al. (2019)	School Health and Reading Program	Oral reading fluency	Grade 2, 3, and 4	Mother tongue (Ateso, Leblango, Luganda, Runyankore, Acoli, Lubgarati, Lumasaaba, Runyoro, Lhukonzo, Lugwere, Lusoga, & Ngakarimojong)
Castillo & Wagner (2019)	Bridges to the Future Initiative	Oral reading fluency	Grade 1, 2, and 3	Mother tongue (Sepedi, Tshivenda & Xitsonga)
Kerwin & Thornton (2020)	Northern Uganda Literacy Project	Oral fluency	Grade 1	Mother tongue (Leblango)
Piper et al. (2016)	PRIMR	Oral reading fluency	Grade 1 and 2	Mother tongue (Lubukusu & Kikamba)
Piper et al. (2018)	PRIMR	Oral reading fluency	Grade 1 and 2	National (Kiswahili); Later acquired (English)
Shin et al. (2019)	Read Malawi	Sentence reading	Grade 2 and 3	National (Chichewa); Later acquired (English)

**Figure 9:**
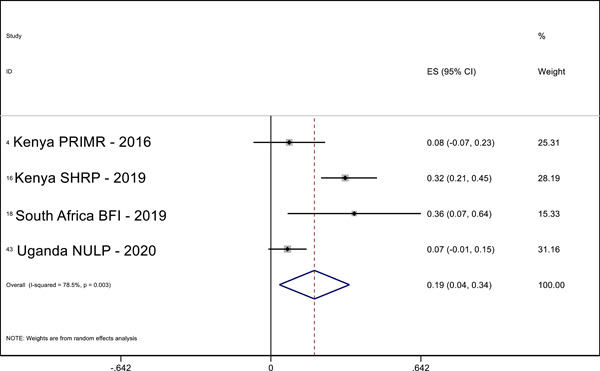
Effects of language of instruction interventions on sentence reading (mother tongue).

Our meta‐analysis examined the estimated effect size of MT teaching and learning materials on sentence reading in the MT. Specifically, we include Brunette et al. (2019), which evaluated the USAID School Health and Reading Program (SHRP) that provided textbooks in MT and English to pupils, accompanying teaching guides, teacher training, and ongoing support to teachers implementing the new program, and Castillo and Wagner (2019), which assessed the Bridges to the Future Initiative (BFI), a program that provided culturally appropriate, interactive language support in three local languages plus English. Results from these two programs along with the NULP and PRIMR were used for our meta‐analysis. We find an estimated effect of 0.19 standard deviations (SMD = 0.19, 95% CI = 0.04, 0.34; *I* ^2^ = 78.5%) of interventions in MT and English language on sentence reading in MT (see Figure 9).

As with other outcomes, we were unable to conduct meta‐analyses for sentence reading in the national language and later acquired language. Instead, we present the effect sizes from each of the individual studies as suggestive evidence of the possible relationship. For the national language, Piper et al. (2018) found a nonsignificant effect size of 0.09 standard deviations (SMD = 0.09, 95% CI = −0.003, 0.18) for the PRIMR program in MT language on Kiswahili sentence reading and Shin et al. (2019) found a nonsignificant effect size of 0.005 standard deviations (SMD = 0.005, 95% CI = −0.10, 0.11) for the Read Malawi program in the national language on Chichewa sentence reading.

#### 8.1.6 Reading comprehension

We were able to include ten studies out of the 29 total quantitative studies which estimated the effects of LOI interventions in MT and national languages on students’ reading comprehension. Of these, five look at reading comprehension in the MT, two look at reading comprehension in the national language, and four look at reading comprehension in the later acquired language. For those studies assessing outcomes in the later acquired language, two studies include student populations that differ from the other studies (Jamaludin et al., 2016; Power et al., 2017). These two studies still focus on primary grade levels, however, Jamaludin et al. (2016) focuses on Grade 6 students (toward the end of primary school as opposed to early grades) and Power et al. (2017) do not state which primary grade levels their study focuses on. Table 10 provides an overview of the studies and how they measured reading comprehension.

**Table 10:** Measurement of reading comprehension.

Study	Intervention name	Definition of outcome variable	Grade level	Language
Brunette et al. (2019)	School Health and Reading Program	Reading comprehension	Grade 2, 3 and 4	Mother tongue (Ateso, Leblango, Luganda, Runyankore, Acoli, Lubgarati, Lumasaaba, Runyoro, Lhukonzo, Lugwere, Lusoga, and Ngakarimojong)
Castillo & Wagner (2019)	Bridges to the Future Initiative	Reading comprehension	Grade 1, 2 and 3	Mother tongue (Sepedi, Tshivenda and Xitsonga)
He et al. (2008)	Pratham PicTalk Program	Aggregated English test score	Grade 2 and 3	Later acquired (English)
He et al. (2009)	Pratham Shishuvachan Program	Normalized total score	Grade 1	Mother tongue (majority Hindi, Marathi and Urdu)
Jamaludin et al. (2016)	Synthetic Phonics	Comprehension skills	Grade 6	Later acquired (English)
		Early reading skills		
Kerwin & Thornton (2020)	Northern Uganda Literacy Program	Reading comprehension	Grade 1	Mother tongue (Leblango)
Lee et al. (2015)	BB Bilingual Schools	Literacy	Grade 4	National (Khmer)
Piper et al. (2016)	PRIMR	Reading comprehension	Grade 1 and 2	Mother tongue (Lubukusu and Kikamba)
Piper et al. (2018)	PRIMR	Reading comprehension	Grade 1 and 2	National (Kiswahili); Later acquired (English)
Power et al. (2017)	English in Action	English language	Primary	Later acquired (English)

**Figure 10:**
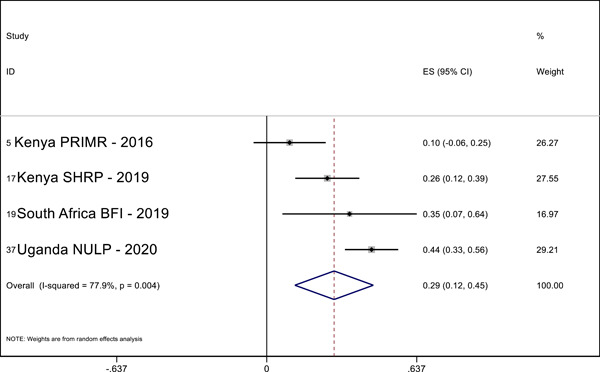
Effects of language of instruction interventions on reading comprehension (mother tongue).

For our meta‐analysis, to determine the effects of LOI interventions in MT languages on students’ reading comprehension, we pool outcomes within studies to generate average effects of interventions on reading comprehension by language type. Four studies (Brunette et al., 2019; Castillo & Wagner, 2019; Kerwin & Thornton, 2020; Piper et al., 2016, 2018) examine interventions which provided new or revised teaching and learning materials in the MT to promote bilingual literacy education. The average effect of MT literacy interventions on MT reading comprehension was estimated as 0.29 standard deviations (SMD = 0.29, 95% CI = 0.12, 0.45; *I* ^2^ = 78%) (Figure 10).

He and co‐authors (2009) assessed the Shishuvachan program, created by the NGO Pratham, which aims at developing reading and comprehension skills through daily, 1‐h long sessions that include seven different activities: pre‐reading, storytelling, story‐reading, word recognition, letter recognition, barakhadi charts, and unfamiliar text reading. In addition, teachers receive special training on the Shishuvachan techniques and classrooms are provided with a child library. He et al. (2009) found an estimated effect of 0.46 standard deviations (SMD = 0.46, 95% CI = 0.25, 0.66) of the Shishuvachan MT intervention on MT reading comprehension.

**Figure 11:**
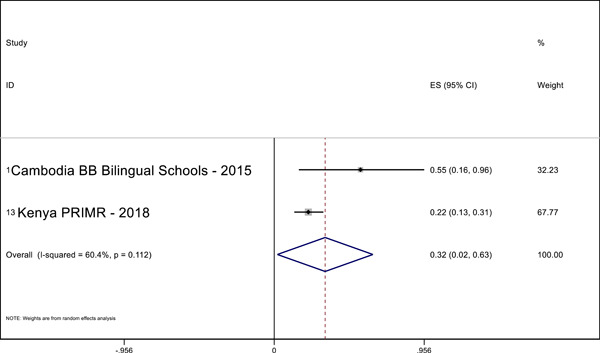
Effects of language of instruction interventions on reading comprehension (national language).

Meanwhile, we conducted a meta‐analysis using the studies of Lee et al. (2015) and Piper et al. (2018). From these studies, we found the average effect of MT instruction on national language reading comprehension was estimated as 0.32 standard deviations (SMD = 0.32, 95% CI = 0.02, 0.63; *I* ^2^ = 60%; evidence from two studies) (Figure 11).

Finally, there are two studies which assess the effectiveness of different types of activities in national and later acquired language on English (or later acquired language) literacy skills (He et al., 2008; Power et al., 2017). He and co‐authors (2008) found an estimated effect of 0.31 standard deviations (SMD = 0.31, 95% CI = 0.12, 0.45) while Power and co‐authors (2017) found an effect size of 0.05 standard deviations (SMD = 0.05, 95% CI = −0.02, 0.11), which was not statistically significant, indicating mixed evidence for the effectiveness of interventions targeting later acquired language literacy.

#### 8.1.7 Writing

Lastly, we examined the effects of LOI intervention in MT and national languages on students’ writing across studies. However, only two of our included studies examine this outcome at all, and the interventions evaluated in these studies are not similar, so our meta‐analysis abilities are limited. Table 11 provides details about the included studies and the outcomes they measured.

**Table 11:** Measurement of writing.

Study	Intervention name	Definition of outcome variable	Grade level	Language
Kerwin & Thornton (2020)	Northern Uganda Literacy Project	Writing score	Grade 1	Mother tongue (Leblango); Later acquired (English)
		Name writing		
Shin et al. (2019)	Read Malawi	Dictation	Grade 2 and 3	National (Chichewa); Later acquired (English)

Only the NULP study examined the effect of the intervention in MT language on writing in the MT and only the Read Malawi study examined writing in the national language. Therefore, we provide the estimated effect size from these studies as suggestive evidence of the overall effect. For MT writing, Kerwin and Thornton (2020) found an estimated effect 0.69 standard deviations (SMD = 0.69, 95% CI = 0.56, 0.81). For national language writing, Shin et al. (2019) found an estimated insignificant effect of 0.05 standard deviations (SMD = 0.05, 95% CI = −0.06, 0.15). More rigorous evidence is needed to determine the effect of LOI interventions on students’ writing.

Both studies examined the effect of their interventions on writing in the later acquired language (English in both cases). Kerwin and Thornton (2020) found an estimated effect of 0.45 standard deviations (SMD = 0.45, 95% CI = 0.30, 0.60) while Shin et al. (2019) found a non‐statistically significant effect of 0.05 standard deviations (SMD = 0.05, 95% CI = −0.06, 0.15).

#### 8.1.8 Publication bias assessment

**Figure 12:**
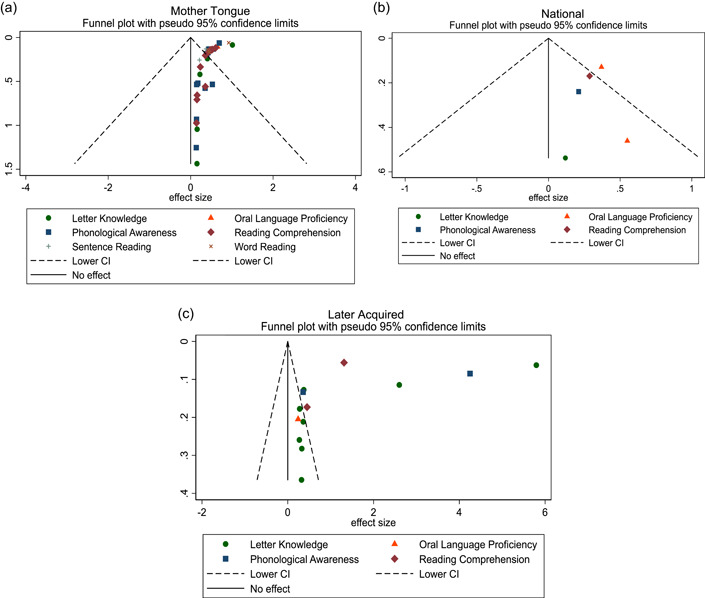
Funnel plots by language type: (a) Mother Tongue, (b) National Language, and (c) Later Acquired Language.

To determine the potential for publication bias in our studies, we generated funnel plots. Using the graphs, we examine the distribution of effects on literacy outcomes by language type. Across language types we find evidence of non‐normal distributions of effect sizes and see results mostly skewed to the right (see Figure 12a–c). Therefore, the results suggest there is likely publication bias in studies that examine the effect of LOI on literacy outcomes whether in the MT, national or later acquired language. This pattern seems consistent regardless of the outcome measured.

### 8.2 Narrative synthesis of quantitative studies

Most of the quantitative evidence suggests that (partial or sole) MT instruction during elementary school has a positive impact on MT literacy outcomes. For example, Piper et al. (2016) ran an RCT to evaluate the PRIMR program in Kenya, which introduced innovative teaching methods, new materials based on the existing Kenyan curriculum, and professional development to build the skills of educators and improve student literacy outcomes. The authors found that the positive impacts on MT literacy were larger when PRIMR included a MT literacy component (PRIMR‐MT) and not just English and Kiswahili (base PRIMR).

Similarly, Walter and Dekker (2011) found that reading ability in MT was about 24% greater when young children (grades 1–3) were taught predominantly in their MT (i.e., Lilubuagen) and learned the language of wider communication (English or Filipino) as a subject. Jain (2017), who investigated the impact of official language policies on education using state formation in India, found that linguistically mismatched districts (i.e., districts where students were taught in a language that differed from their MT) had 18% lower literacy rates than districts where the LOI was the MT of most students. By leveraging the 1956 reorganization of Indian states along linguistic lines and implementing a district‐level difference‐in‐differences (DID) approach, Jain (2017) found that literacy rates increased by 37% after the 1959 reassignment that led to MT instruction.

A similar strategy was used by Argaw (2016), Ramachandran (2017) and Chicoine (2019), who all leveraged Ethiopia's introduction of MT‐based education in primary school during the 1990s to estimate its effect on literacy via three distinct approaches: DID, triple‐differences, and DID with continuous treatment, respectively. Although they focused on the language policy and used similar binary outcomes, their combined evidence was inconclusive: on the one hand, Argaw (2016) and Ramachandran (2017) found that access to MT schooling increased reading ability; on the other, Chicoine (2019) reported null effects of MT instruction on the ability to read and write.

Null impacts of MT instruction on MT literacy were also reported by Hynsjo and Damon (2015), who found weak and inconclusive evidence that indigenous children who attended Quechua‐medium schools attained higher language test scores in Peru. The authors did, however, caution about the limitations of their study for providing causal evidence. They estimated impacts through multivariate regressions using cross‐sectional Peruvian school‐level data from the Young Lives international study of childhood poverty, hence their strategy was prone to selection bias.

Some of the studies also provided evidence on how MT education affects literacy in the later acquired language. Most of the evidence suggests that MT instruction has null impacts on the literacy of the later acquired language. For instance, Piper et al.'s 2016 experimental evaluation of the PRIMR intervention showed that assignment to the MT group had no additional benefits for English or Kiswahili learning outcomes beyond the non‐MT group (also receiving the base PRIMR intervention). Null impacts of MT instruction on English were also reported by Laitin et al. (2019). Their study assessed the effects of instruction in Kom (a local Cameroonian language) on English literacy by comparing student test scores between 12 treatment schools with MT LOI during the first 3 years of primary schooling, against 12 control schools where English was the sole LOI.

In contrast, Taylor and Coetzee (2013) found that MT instruction in grades 1‐3 was correlated with better English literacy in grades 4–6. Their study compared test scores of South African children who attended a school where MT was the LOI during the first years of primary with scores from students who went to institutions where English was the LOI since first grade. In line with this result, Walter and Dekker's 2011 study in the Philippines (cited previously) found that children receiving most of their education in Lilubuagen their MT, performed consistently better in English and Filipino than those who were taught solely in these latter two languages.

Two other studies look at the impact of MT instruction on LWC's literacy in contexts where English is not the lingua franca but do not find any effects. Lee and co‐authors (2015) found no differences in Khmer literacy (Cambodia's official language) between students from an ethnic minority attending bilingual schools and students from the same ethnic minority who were only instructed in Khmer (i.e., attending monolingual schools). Santibanez (2016) compared literacy outcomes between indigenous students attending bilingual schools—where they were supposed to be taught in their MT from kindergarten through 3rd grade before gradually making a full transition into Spanish by the 6th grade—and indigenous students attending schools in Spanish. She found null impacts of bilingual schools on Spanish literacy.

The next set of studies assessed the effectiveness of methods or interventions aimed at increasing literacy either in MT or in a second or third language. These studies evaluated programs that provided teacher training and support, learning inputs such as books and textbooks translated to MT, computer‐assisted learning activities, and new curriculums, among others. Overall, the evidence rendered by this group of studies suggests that providing school inputs (such as textbooks and other teaching materials) together with teacher training improves MT literacy outcomes in children attending elementary school. However, some of these papers also highlighted important limitations that may hinder positive impacts, such as orthographic complexity of local languages, implementation challenges, non‐linear teaching productivity, and complementarity of inputs.

Sailors et al. (2010) assessed the READ intervention in South Africa, which distributed high‐quality children's books both in English and 10 different MTs (depending on the school's LOI) among students in grades 1 and 2. In addition, the program provided teachers with strategies for engaging students in meaningful encounters with those books. Their findings suggest that children in treated schools performed significantly better both in their MT and in English (their second or third language). In the same vein, Brunette et al. (2019) found that MT print‐enriched environments jointly with complementary teacher trainings positively affected MT literacy. The authors evaluated the SHRP that provided textbooks in MT and English to pupils, accompanying teaching guides, teacher training, and ongoing support to teachers implementing the new program. Their results indicate that the SHRP program increased mother‐tongue literacy; moreover, in contrast with Sailors et al. (2010), this study estimated literacy gains for each local language, finding that the magnitude of the impact varied by MT. Specifically, SHRP increased literacy achievement in 9 of the 12 local languages, having no significant impact in the three most orthographically complex Ugandan languages.

Lucas et al. (2014) also found significant positive impacts of a program combining both training and mentoring to early‐grade teachers provisioned with literacy materials in the official LOI and English. They evaluated the same intervention in both Kenya and Uganda and found that while Ugandan literacy (in Lango) increased, Kenyan literacy (in Swahili) increased to a smaller degree. Students in Kenya were tested in Swahili, which is not necessarily the main LOI in primary schools, despite official policy. In addition, it is noteworthy to point out that many Kenyan students spoke a MT similar to Swahili, but not necessarily with a formally established orthography. Hence, differences in impacts between countries could also be due to differences in the percentage of students for which Lango or Swahili was their MT. Unfortunately, the evaluation data did not include student‐specific data on MT.

Simsek and Alisinangolu (2008) were also interested in the impact of MT interventions on the literacy outcomes of pre‐school children. The intervention they studied involved storytelling along with before‐story, during‐story, and after‐story activities. The results indicate that the program had a positive impact on the reading readiness and overall literacy of Turkish pre‐schoolers in their sample.

Lastly, two papers in this group studied the effects of other types of literacy interventions that did not involve the joint provision of teacher trainings and learning materials. For example, Wawire and Kim (2018) used a RCT to estimate the impact of an 8‐week additional instruction program on phonological awareness and letter knowledge in Kiswahili, the MT of over 70% of the Kenyan children in their sample. They found that supplemental training in Kiswahili yielded large effects (ES from 0.37 to 0.95 SMD) in phonological awareness and letter‐sound knowledge both in Kiswahili and English. They also reported null impacts on reading outcomes across both languages.

Castillo and Wagner (2019) evaluated the impact of a 2‐year multimedia reading program for rural South African children in grades 1–3. The authors found that children in the treatment group demonstrated nearly three‐quarters of a year of additional reading growth and a twofold increase in reading comprehension compared with children in the control group. The program provided culturally appropriate, interactive language support in three local languages plus English. The activities emphasized by the instructional software were phonemic awareness, listening, sentence construction, typing and spelling, grammar and punctuation, and short passage reading and comprehension.

Lastly, there are several studies that assessed the effectiveness of different types of activities at increasing literacy in English as a second (or third) language. Most of these studies reported positive impacts of different types of interventions designed to improve literacy in English. He et al. (2008) evaluated the Pratham English Language Program for grades 1–5 in India. The intervention included both a machine‐based implementation strategy and activities based on flashcards and teacher manuals. The authors findings show that all implementation strategies positively affected students’ knowledge of English. Weaker students benefitted more from interventions that included teacher implemented activities while higher performing students gained more when they are allowed to self‐pace their learning through the machine‐based only intervention. The authors designed two different RCTs to assess the relative effectiveness of different implementation methods of the English program. The experimental designs varied the implementation technology and whether the intervention was delivered through externally hired tutors or public‐school teachers.

In contrast with the previous evidence, Power et al. (2017) found that an English language development project in Bangladesh that provided classrooms with audio resources and trained teachers on new activities and approaches had no effect on English proficiency. According to their DID estimates, the intervention had null impacts on the share of students achieving the lowest grade in the Graded Examinations in Spoken English (GESE) test across primary and secondary school.

### 8.3 Synthesis of qualitative studies

#### 8.3.1 About the interventions

The qualitative studies selected as high quality for this systematic review include studies from Timor‐Leste, the Philippines, Kenya, India, Cameroon, Rwanda, Nepal, and Mozambique. The studies were published between the years 2000 and 2019. Four of the eight studies, those in Timor‐Leste, the Philippines, Kenya, and India, explored MT education interventions. The study conducted in Mozambique explored a bilingual intervention that included instruction in the students’ MT and in Portuguese. The remaining four studies in Cameroon, Rwanda, and Nepal investigated LOI in English as a later acquired language (called English Medium Instruction or EMI in the following sections). All of the programs that were either evaluated or investigated in these studies were school‐based programs and all data collection took place within the school settings.

#### 8.3.2 Findings about the methods

Half of the studies included in this systematic analysis are purely qualitative studies in the form of case studies. They include methods such as focus group discussions with children, key informant interviews with provincial level stakeholders, parents, teachers and children, classroom observations, and the analysis of written artifacts from teachers and students such as student homework and teacher corrections. The remaining 6 studies in this systematic analysis were mixed methods analyses. Quantitative data collection across the six mixed method studies consisted of administering the ERGA, administering written tests in school subjects such as the MT and second or later acquired languages and mathematics and natural sciences, vocabulary, and comprehension tests, monitoring enrollment and attendance. Qualitative methods across the six mixed methods studies included classroom observations, focus group discussions with students, and teacher interviews. The below findings and analysis focused on the purely qualitative studies and on the findings obtained from the qualitative methods used in the mixed method studies.

#### 8.3.3 Findings about the target populations

All of the eight high quality studies except for Sah and Li's (2018) study in Nepal, focused on primary schools, including students from kindergarten to Grade 6. Sah and Li, however, examined a secondary school, including students in Grade 7 and Grade 9. Two studies took place at rural schools, one took place in a suburban school that attracted many migrants from rural areas, two studied urban schools, and one analyzed a program in both urban and rural school. Two studies did not specify if the schools were in urban or rural areas.

#### 8.3.4 Findings about the activities

Across the bilingual instruction and EMI interventions analyzed across the studies, one of the common main activities was the presence and usage of quality teaching and learning materials in the MT for adequate learning. Parents of students also indicated that lack of materials was a barrier for child's learning (Mose, 2016). Researchers perceived that the lack of materials affected teachers’ performance, as well. Teachers were perceived to be enthusiastic and have positive attitudes on implementation of an MT initiative when there was availability of teaching materials in the MT language. Additionally, the studies indicated that the adapted learning and teaching materials were more helpful if the materials were provided on time (such as during implementation of MT based program or during transition) and if the materials included cultural nuances and realties of students’ learning capacity in classrooms. Regarding the timing of provision of adapted learning and teaching materials—if students and teachers received materials after implementation of a MT based program, both teachers’ ability to support students and students’ enthusiasm decreased (Caffery et al., 2016; Harden et al., 2020; Milligan et al., 2016). Further, a study conducted in Rwanda found that materials were more usable and accessible when it included easier English content and provided a glossary in the MT language (Milligan et al., 2016). In essence, the teaching and learning materials were perceived as more effective and helpful if they were of high quality – accessible in the MT language, provided on time, considered the cultural contexts, and included glossaries using the MT language—rather than when they were low quality – not accessible in the MT, delivered late, and ignored the local context.

Our analysis also showed the importance of having context‐relevant materials. For example, commenting on the importance of teaching materials which reflect the realities of the classrooms, a teacher in Rwanda remarked; “The intervention textbooks have short texts, and this makes it easy to understand the content…The use of Kinyarwanda also helps in the understanding of the content, but the other books (do not) have this aspect and learners had to struggle to follow the teacher” (Milligan et al., 2016). Similarly, a study in India indicated that teachers found materials with culturally appropriate content easier to use and allowed children to be more engaged. The materials included stories related to community life and had corresponding illustrations (National Council of Educational Research and Training NCERT, 2011). Some activities also included capitalizing on social context to encourage the use of the students’ MT using Basic Interpersonal Communication Skill (BICS) (National Council of Educational Research and Training NCERT, 2011). The NCERT team explained that BICS, combined with teaching academic content, is an activity that provided “a space for children to learn within their cultural context” (National Council of Educational Research and Training [NCERT], 2011).

Our analysis also suggests that provision of quality teacher training was another main LOI transition activity. Teacher trainings across studies were provided through various platforms such as workshops, one on one coaching, training‐of‐trainers models, field visits to classrooms, teacher guides or student‐centered teaching strategies. To deliver high quality teaching, student centered teaching methodology was also included as part of training materials and workshops for teachers (Caffery et al., 2016). Further, a pioneer program in Timor Leste used pedagogic principles outlined in the country's national policy to develop teacher training materials (Caffery et al., 2016). In addition, an activity employed in the EMI studies was teacher training in language supportive pedagogy wherein Math, Social Studies, and Science teachers were trained on how to use language supportive textbooks and associated teacher guides in the classroom (Milligan et al., 2016). A study in Mozambique conducted by Benson (2000) also indicated that teachers received mini training seminars to enhance teacher training. Teachers were trained by teacher trainers on various components ranging from MT linguistics, teaching strategies, and literacy but teachers were not provided with adequate teaching methods (Benson, 2000). The study highlighted that training for teachers need to be more goal orientated. Further, data from a study conducted in Orissa, India suggested that utilizing teacher training materials in teachers’ languages, inclusion of lesson planning strategies and addressing teachers’ concerns in a timely manner during the training sessions had the potential to enhance quality of teacher training (National Council of Educational Research and Training [NCERT], 2011). Across the studies, we see that the lack of adequate teacher training and teacher support was a major barrier to students’ learning. For example, data from a study in Nepal suggested that teachers did not have capacity to do language correction and made errors when correcting students’ work, highlighting the importance of providing adequate training focused on pedagogical skills for teachers to support students (Sah & Li, 2018). Other activities described in the studies explored the introduction of the MT into the classroom, including structured bilingual school schedules (Benson, 2000), and introducing MTE‐specific curricula (Caffery et al., 2016).

#### 8.3.5 Findings about outputs

Primary outputs of two MT studies, Caffery et al. (2016) and National Council of Educational Research and Training (NCERT) (2011), included Improved Curriculum outputs, whereas we found no Improved Learning Standards or Improved Assessments outputs found in the analysis of the qualitative or mixed methods studies. Positive “Improved Curriculum” outputs include bilingual materials being displayed and used in classrooms and an overwhelming positive reception and wide support of MT‐specific learning materials such as textbooks by students and teachers alike (Caffery et al., 2016; National Council of Educational Research and Training [NCERT], 2011). Studies that focused on improving curriculum and learning resources often reported that the bilingual learning materials were hard to come by, either due to a reliance of production on international organizations or due to lack of local dissemination infrastructure to reach rural schools (Caffery et al., 2016; National Council of Educational Research and Training [NCERT], 2011). Our analysis suggests that the lack of sustainable infrastructure to produce and disseminate learning materials often act as barriers to full implementation of MT programming.

#### 8.3.6 Findings about outcomes

##### Intermediate outcomes

Intermediate outcomes of the MTE suggested positive perceptions with regard to teaching quality and increased student motivation. For example, teachers started using more activity‐based teaching (National Council of Educational Research and Training [NCERT], 2011). Regarding student motivation, teachers reported observing increased student self‐esteem (National Council of Educational Research and Training [NCERT], 2011) and increased student engagement and excitement in the classroom (Benson, 2000). In fact, Benson (2000) found indications that students in the MT program seemed to be “significantly different [from students not in the program] in terms of liking school, helping even older siblings with schoolwork, and taking the initiative at home.”

The three positive intermediate outcomes noted in the ToC were only observed in Milligan, Clegg and Tikly's (2016) English‐medium intervention study in which they studied the quality of learning and teaching in Primary 4 English‐medium classes across Rwanda. These included improved quality of teaching where the majority of teachers and students were observed actively using textbooks during class, compared to a baseline of only 25% of teachers doing so (Milligan et al., 2016). Positive student motivation outcomes included increased student engagement and excitement inside and outside of the classroom (Milligan et al., 2016). Additionally, parents’ involvement in student learning increased when students were able to take their books home and continue learning in the household (Milligan et al., 2016). No other English‐medium intervention study reported positive intermediate outcomes.

Negative intermediate outcomes on teacher and student motivation were only reported in one English‐medium intervention study. Mose (2016) found that in Kenyan classrooms that used Kiswahili and English to teach, students interviewed stated that though they were unable to understand English, teachers continued to use English, hindering students’ capacity to learn and to have positive teacher‐student interactions. Further, teachers themselves indicated that students found English difficult to understand. Students’ lack of understanding effected teacher's motivation (Mose, 2016, 2016). Students’ lack of understanding effected teacher's motivation (Mose, 2016). When interviewed, teachers cited students’ inability to master English quickly enough to be able to read on their own as extremely discouraging. Furthermore, classroom observations also showed little‐to‐no responses from students in classroom interactions (Mose, 2016).

##### Final outcomes

Positive final outcomes from MTE studies include perceived improvements in MT and second or later acquired language reading skills, especially in comparison to students who were not studying in the MTE schools (Benson, 2000; National Council of Educational Research and Training [NCERT], 2011). This could be related to the fact that teachers seem to be open to using MT in the classroom, even if there might be a negative stigma around it or if they disagree with the MTE policy (Harden et al., 2020). Other positive outcomes reported, which are not reflected in the ToC, include parents who were happy that traditional cultural practices were being promoted in the schools (Benson, 2000), and increase in enrollment, better retention rates, and lower drop‐out rates in MTE schools (National Council of Educational Research and Training [NCERT], 2011). There were no reports of the children's MT oral skills improving in any MT studies. Unexpected negative outcomes included children did not have the same MT as the majority of students in the schools leaving the schools, a preference for Oriya over the MT, and decreased participation of disabled children (National Council of Educational Research and Training [NCERT], 2011).

Positive final outcomes from the English medium programs included evidence that suggests language supportive learning may have had positive effects on learner second or later acquired language reading and oral skills (Milligan et al., 2016) and increased confidence speaking English (Sah & Li, 2018). Negative outcomes included a possible loss in reading and writing in the learners’ MT while the students were also not succeeding in learning English or the content they were being taught in English (Mose, 2016; Sah & Li, 2018). As Sah and Li (2018) articulated it, switching to an English medium model “without enough preparedness contributed to a comprehension crisis in content learning, low proficiency in both English and Nepali, and loss of MT for the students, resulting in wider achievement gaps between the rich and the poor.” Finally, poor learning outcomes and poor teacher morale were observed in English medium schools due to lack of comprehension (Mose, 2016).

#### 8.3.7 Assumptions, potential moderators, and possible facilitators and barriers

**Moderators**: Using the “best fit” synthesis framework, our analysis of the data across the studies provided little to no information regarding the moderators as outlined in the ToC. Data to match with the potential moderators under the “individual” category in the ToC were limited as the studies did not consider student's characteristics such as age, sex and disability status and its input on learning. Similarly, regarding the “School and Institution” category, only one paper mentioned the lack of proper physical infrastructure such as running water and electricity in MTE schools, although it did not outline how infrastructure could influence MT‐based learning versus another medium of instruction (National Council of Educational Research and Training [NCERT], 2011). The paper highlighted the variations in availability of infrastructure supports between different MTE schools. For example, data found that running water was available in all Kuvi‐language and Santhali‐language schools but only in 11% of Bonda‐language schools (National Council of Educational Research and Training [NCERT], 2011). This observation could indicate that some tribal languages in India have more political support than others.

Another factor from the studies which is related to the “Teacher Training” factor listed as a potential moderator in the ToC can be attributed to the additional support for teachers provided through senior teachers, language coordinators or having two teachers in one class. Dedicated language coordinators helped increase teachers’ confidence in dealing with new and complex realities of classrooms using a new program (Caffery et al., 2016). Interaction between two teachers in one class helped increase students’ enthusiasm to participate in classroom activities (Caffery et al., 2016).

**Possible facilitators**: Our analysis shows that community buy‐in can be considered a key facilitator as it relates to the factors of “political will and local policy,” and “community language use” listed in the ToC. Caffery et al. (2016) found that implementation of community advocacy campaigns on the MTE program in Timor Leste led to the community welcoming the program warmly. Further, inclusion of community‐based contextual information into the new language curriculum such as local stories from community members was seen as positive ways to make the curriculum rich and engaging to children studying that MT. Community members were also seen narrating the local stories from the curriculum to students (Caffery et al., 2016). The same study also hinted that having parental involvement and buy‐in (which are factors related to “Parental language demands” as listed in the ToC) helped increase parents’ engagement with their children regarding education activities such as increased homework support. Similarly, an MT program in Orissa, India specifically included associations such as Parent‐Teacher associations and Village Education Committees to facilitate the implementation of the MT program through community involvement (National Council of Educational Research and Training [NCERT], 2011). Community members assisted in material development, and participated in traditional games and arts (National Council of Educational Research and Training [NCERT], 2011).

A key assumption mentioned in the ToC is “Teachers speak the language” and our analysis found that in studies where teacher implementation of the intervention is high, a common factor is that teachers’ native language corresponded to the MT used to teach. For example, Mose (2016) found that more than 90% of the teachers in the intervention region of Rwanda were native Ekegusii language speakers which assisted in high teacher implementation of the intervention. Similarly, a study in Philippines found that one of the main factors in teachers’ higher rates of implementation of a MT policy was if teachers spoke the same MT, felt very comfortable in using the MT and believed that the MT usage was one of the best tools for children's literacy acquisition (Harden et al., 2020). A teacher's high fidelity implementing the MT policy was driven by the teacher's own linguistic background and usage of the MT. Adding to the importance of teachers having knowledge of MT, observations from a team of researchers found that the continued use of MT by teachers even outside of the classroom showed teachers’ commitment to using child‐centered pedagogy principles (Caffery et al., 2016).

### 8.4 Integrated synthesis

In the integrated synthesis, we draw on the findings of the meta‐analysis, the findings from the quantitative narrative synthesis, as well the qualitative synthesis to provide a cohesive picture of the impact of LOI programs on literacy outcomes, the intermediary outcomes of quality of teaching and learning materials, teacher quality, student motivation, and parental and community engagement in literacy learning.

The findings from the quantitative and qualitative studies generally point toward MT instruction having a positive impact on MT literacy outcomes (Argaw, 2016; Jain, 2017; Ramachandran, 2017; Piper et al., 2016; Walter Dekker, 2011). Although there were two studies that showed no significant impacts (Chicoine, 2019; Hynsjo & Damon, 2015), the evidence from the qualitative synthesis suggests that quality of instruction—especially tailored to a structured pedagogy approach for that language—is critical in ensuring a MT program has a significant impact literacy acquisition on MT language literacy outcomes.

In terms of the types of inputs that are most likely to impact within language learning outcomes, i.e., MT inputs on MT outcomes and later language inputs on later language outcomes, both the quantitative and qualitative studies showed that high‐quality, structured textbooks along with continuous teacher support on how to engage meaningfully with the books (Brunette et al., 2019; Caffery et al., 2016; Harden et al., 2020; Lucas et al., 2014; Milligan et al., 2016; Sailors et al., 2010; Simsek & Alisinangolu, 2008) were critical variables in impacting within language literacy outcomes.

There were some clear variables that emerged from both quantitative and qualitative studies as those moderating the impact of LOI interventions on literacy outcomes. First, was the nature and complexity of the MT's orthographic properties (Brunette et al., 2019). This is critical given that there may not be a one‐size‐fits‐all for MT program development, and that language‐specific pedagogy is important in the development of a MT program (Benson, 2000; Caffery et al., 2016). Second is political will, community demand for each language, and the status of each language in the community and the educational system (Caffery et al., 2016; Harden, Sowa, & Punjabi, 2020; National Council of Educational Research and Training [NCERT], 2011). The third factor related to the importance of having contextually relevant teaching and learning materials (National Council of Educational Research and Training [NCERT], 2011). Lastly, teachers’ own linguistic backgrounds were significant in determining fidelity of implementation of the MT programs (Harden et al., 2020; Mose, 2016).

The evidence on MT instruction on the later acquired language paints a more mixed picture from both the quantitative and qualitative studies. Some quantitative studies showed no significant impacts of MT or bilingual instruction on later acquired language literacy outcomes (Laitin et al., 2019; Lee et al., 2015; Piper et al., 2016; Santibanez, 2016); while others showed positive associations between MT programs and later acquired language literacy outcomes (Taylor & Coetzee, 2013; Walter & Dekker, 2011). In the qualitative studies, only one study demonstrated positive outcomes on English literacy outcomes in an English as a second language program (Milligan et al., 2016); however, it showed the critical importance of the quality of the teacher and learning materials and teaching. Other studies reiterated this by showing that English instruction too early or with poor quality only diminished motivation and learning outcomes further (Mose, 2016; Sah & Li, 2018). The evidence is still inconclusive on how much MT instruction can support English (or later acquired language) relative to high quality teaching in the later acquired language.

In looking at the components of literacy outcomes in line with the CFRA (Hoover & Tunmer, 2020) in our theory of change, the quantitative studies illustrate that there are relatively strong impacts of MT instruction and MT materials on MT learning outcomes, especially word reading (Kerwin & Thornton, 2020; Piper, 2018); phonological awareness (Kerwin & Thornton, 2020) and reading comprehension (Brunette et al., 2019; Castillo & Wagner, 2019; He et al., 2009; Kerwin & Thornton, 2020; Piper et al., 2016, 2018). There is also some evidence for impacts of English or later acquired language interventions on English or later acquired language outcomes (Piper et al., 2018). The evidence for impacts across languages, that is, an intervention in a MT impacting literacy outcomes in a second language, provided more mixed results, with the primary positive impact being in phonological awareness and letter naming (Wawire & Kim, 2018). No significant impact was found for across language oral language outcomes (Piper et al., 2018; Shin et al., 2019). These results are in line with previous reviews on the impact of MT education on MT reading outcomes (Evans & Acosta, 2020; Nag, Vagh, Dulay, Snowling, 2019) and bilingual reading research that shows that oral language skills are far less susceptible to transfer than script‐processing skills (Chung et al., 2019).

## 9 DISCUSSION

### 9.1 Summary of main results

Overall, we identified 45 relevant studies to include in our final review and analysis. For quantitative studies, we conducted meta‐analyses by literacy skill measured. We find positive effects of LOI interventions on letter knowledge, word reading, sentence reading, and reading comprehension in the students’ MT, positive effects on word and sentence reading, and reading comprehension in the national language, and oral language proficiency, word, and sentence reading, reading comprehension, and writing in the later acquired language. We further find null effects of MT instruction on letter knowledge for the national language in Kenya PRIMR and a negative effect on letter knowledge for the later acquired language in Read Malawi using narrative quantitative synthesis. However, our results suggest publication bias in studies that examine the effect of LOI on literacy outcomes regardless of the language or outcome measured.

We also conducted a narrative synthesis of the eligible quantitative studies. Most of the quantitative evidence suggests that (partial or sole) MT instruction during primary school has a positive impact on MT literacy outcomes. Some studies also provided evidence on how MT education affects literacy in the later acquired language, though, most of the evidence suggests that MT instruction has null effects on later acquired language literacy outcomes. The evidence also suggests providing school inputs (such as textbooks and other teaching materials) together with teacher training improves MT literacy outcomes for children attending primary school. Even so, the evidence also highlighted potential limitations to the effectiveness of these programs, such as the orthographic complexity of local languages, implementation challenges, non‐linear teaching productivity, and complementarity of inputs. Lastly, there are several studies that assessed the effectiveness of different types of activities at increasing literacy in English as a second (or third) language. The majority of these studies reported positive impacts of different types of interventions designed to improve literacy in English.

Results from our qualitative meta‐narrative suggest the main activity necessary for a successful LOI program is the presence of high‐quality teaching and learning materials in the MT. Moreover, primary outputs of the MT studies were perceived to be related to improved curriculum including bilingual materials being displayed and used in classrooms, and positive perceived reception and wide support of MT‐specific learning materials such as textbooks by students and teachers alike. Intermediate outcomes of MTE indicate positive attitudes to teaching quality and increased student motivation while final outcomes from MTE studies include perceived improvements in MT and second or later acquired language reading skills, especially in comparison to students who were not studying in the MTE schools. Positive final outcomes from the English medium programs included evidence that suggests language supportive learning may have positive effects on learner second or later acquired language reading and oral skills and increased confidence speaking English. Negative outcomes include a possible loss in reading and writing in the learners’ MT while the students were also not succeeding in learning English or the content they were being taught in English.

### 9.2 Overall completeness and applicability of evidence

We were able to find studies to match all our inclusion criteria, although not every LMIC was included. This naturally limits the generalizability of the findings across all LMICs. Our review also indicated that the outputs of improved curriculum and improved learning standards were not included in any of our included studies, indicating the possibility that the path from LOI transition activities to reading outcomes is more likely to be through the intermediate outcomes of improved teaching quality reflecting a theory of language‐specific learning and bilingual and multilingual learning, an increased motivation to learn, and more engaged communities. We do underscore the importance of being cautious about the generalizability of this finding given the small number of studies that were finally included in our analysis.

### 9.3 Quality of the evidence

Our systematic review applied a rigorous, multi‐step process to ensure the quality of the studies that were finally reviewed for this study. Most included quantitative studies used experimental or quasi‐experimental designs, though seven non‐experimental studies were also included. These seven studies were non‐experimental cross‐sectional and longitudinal studies assessing changes in literacy outcomes at two different time points without accounting for the counterfactual or other confounding factors. The evidence from these studies is, therefore, of a lower quality as they do not provide unbiased impact estimates; however, the results from these studies are still important for understanding relationships between LOI policies and literacy outcomes, and highlight where more research is needed.

In this process it was also clear that there are a considerable number of studies that are of low quality in terms of risk of bias, and more critically there are very few studies that examine transition issues or cross‐language transfer issues in multilingual education settings in LMICs.

Of the final 29 quantitative studies, nine studies were rated low risk of selection bias, 19 were rated as having a low risk of performance bias, 11 included quantitative studies were rated as having a low risk of outcome and analysis bias, and 12 were rated as having a low risk of other biases. As such, we only had a few studies to conduct a meta‐analysis, and were not able to make any conclusive claims about the impact of transition per se.

Of the final 16 qualitative studies, eight emerged as high quality. These eight high quality studies were conducted in eight different countries across three regions: Cameroon, Mozambique, Rwanda and Kenya in sub‐Saharan Africa, Timor Leste in the Pacific region and Nepal, India, and Philippines in Southeast Asia. Even this set of studies only allowed us to make claims regarding the factors that support MT education or EMI education, but there were not enough studies of quality to conclusively discuss transition models’ effectiveness.

### 9.4 Limitations and potential biases in the review process

This study had a few limitations that are important to keep in mind to interpret the results carefully. Importantly, there were limited studies—both quantitative and qualitative—that were considered rigorous enough to be included in our final analyses. For the quantitative analyses, this made it very difficult to extract relevant information on effect sizes and standard deviations in the meta‐analyses. There was also limited overlap in outcome measures, rendering it difficult to pool effects from different studies in the meta‐analyses. Furthermore, the presence of publication bias raises questions related to what extent the findings of this review are generalizable. For the qualitative analyses, within the few studies, we had little information on the breakdown of literacy outcomes, making it hard to triangulate the information across quantitative and qualitative studies.

### 9.5 Agreements and disagreements with other studies or reviews

The study found positive impacts of MT pedagogical inputs on MT literacy outcomes, which is in line with previous systematic reviews (Evans & Acosta, 2020; Nag, Vagh, Dulay, Snowling, 2019).

We were unable to ascertain the impact of transition itself on literacy outcomes. While this neither corroborates nor contradicts previous research, it does raise a question of how the findings square with the multiple studies that show that MT reading is one of the strongest predictors of later acquired language reading (see August & Shanahan, 2010; Chung et al., 2019; Koda & Reddy, 2008 for reviews). Given that most of this evidence is correlational and that we did not have enough studies of quality to answer this question, the answer to the question on the impact of transition on literacy outcomes and how such impacts differ by contextual characteristics requires more research.

## 10 AUTHORS’ CONCLUSIONS

### 10.1 Implications for practice and policy

Our evidence synthesis has various implications for practice and policy. First, quantitative evidence indicates that children are most likely to learn to read in their own MT or in a language they understand best first. In other words, MT‐based LOI interventions may improve students’ letter knowledge, word reading, sentence reading, and reading comprehension in the students’ MT, improve students’ word and sentence reading, and reading comprehension in the national language, and improve students’ oral language proficiency, word, and sentence reading, reading comprehension, and writing in the later acquired language. The evidence highlights that this is likely to be the case only when there are high‐quality materials; in one study the higher quality program was linked to higher cost, however. Further, the evidence indicates that other factors such as political status of the language and orthographic and linguistic type are critical, and programming should take into consideration the sociolinguistic and psycholinguistic milieu of the context before development.

The quantitative studies further indicate that decision makers can invest in high‐quality curriculums and teacher training in the MT. The quantitative narrative synthesis suggests that providing school inputs (such as textbooks and other teaching materials) together with teacher training improves MT literacy outcomes in children attending primary school.

The qualitative results showed that high‐quality inputs in programs for English as a later acquired language were supportive of perceived gains in English language skills. However, given the lack of conclusive evidence on the trade‐off between putting resources in high‐quality English medium education versus high quality MT education and then transitioning to English medium education, more research is needed before providing specific guidance on how to effectively construct transition policies and programs.

In a similar vein, there were no conclusive results from either the quantitative or qualitative studies—and large evidence gaps—related to the impact of first language or MT education programming on second language literacy outcomes post transition. While we can say that the likelihood of literacy outcomes is stronger in the MT when instruction begins in the MT, there are still major evidence‐gaps on how to design programs to effectively transition to a new LOI.

### 10.2 Implications for research

As indicated by the implications for practice and policy, our evidence synthesis highlighted that there are significant evidence gaps in LOI issues in basic education. Therefore, the need for further research in this area is urgent, especially studies that can correct for selection bias, spillovers, and are consistent with various reading outcomes, especially those pertinent to different orthographies (such as different degrees of orthographic depth or tonality, for example) and those pertinent to second and later language learners (such as metalinguistic skills, for example). The included quantitative studies showed significant biases, including publication bias and there is a need for more studies that address the evidence‐gap and a need for studies to be pre‐registered to limit publication bias. In the qualitative studies as well, there were many studies that had nebulous research objectives and questions, and a number that had methods and made claims that were not aligned with their research questions—leading to conclusions that may not be valid.

The key area for future research for LOI transition would be to investigate with high quality studies whether—and if so, in what ways and why—MT education programs impact literacy and other academic outcomes in not only the MT, but also in the national languages and in post‐colonial international languages. Furthermore, cost‐effectiveness analyses are critical to help provide decision makers with the trade‐offs that are inherent in determining how much time and resources should be spent teaching in one language versus another. Researchers should work with education decision makers across LMICs, such as decision‐makers on Ethiopia's new Education Roadmap, Senegal's new Language of Instruction policy, and Mozambique's planned new bilingual education policy, to design rigorous mixed‐methods experimental or quasi‐experimental studies that respond to key decisions of policymakers by carefully examining impacts of LOI policies within languages (impact of MT programming on MT outcomes, and impact of later acquired language programming on later acquired language outcomes) and across languages (impact of MT programming on later acquired language outcomes). Such research should include cost‐effectiveness analyses to enable policymakers to make decisions about investments in curriculum design and teacher training.

Another important implication for research was that there was unclear and limited information on the nature of the literacy and language constructs that are under examination. This was more starkly the case in the qualitative and mixed‐methods study where “reading” and “language” are treated as singular constructs. This makes it hard to triangulate information across studies, but also within studies it makes it difficult to make practical implications on how the study results can be used to design better programs or policies. Foundational literacy acquisition is a multi‐dimensional, progressively changing skill—and the more decomposed skill information that is available the more applicable the results.

Finally, there is still relatively limited focus of geographic reach of the studies. The nature of language differences, linguistic compositions, politics of LOI policy implementation, and degree of linguistic heterogeneity are different across countries, and contexts within countries, and it would be valuable to have studies from across a wider range of geographies to understand the generalizability of LOI policy effectiveness.

## ROLES AND RESPONSIBILITIES

- Content: Pooja Nakamura, Zelealem Leyew
- Systematic review methods: Thomas De Hoop
- Statistical analysis: Adria Molotsky, Rosa Castro Zarzur
- Qualitative analysis: Varsha Ranjit, Yasmina Haddad
- Information retrieval: Liz Scalia

## SOURCES OF SUPPORT

Describe the source(s) of financial and other support for the proposed review.

## DECLARATIONS OF INTEREST

The authors have no vested interest in the outcomes of this review, nor any incentive to represent findings in a biased manner.

## PLANS FOR UPDATING THE REVIEW

The lead author, Pooja Nakamura, will update the results of this systematic review if funding is available.

## Supporting information

Supporting information.

## Footnotes

1 Learning science is the field of study that aims to “better understand the cognitive and social processes that result in the most effective learning, and to use this knowledge to redesign classrooms and other learning environments so that people can learn more deeply and more effectively (Sawyer, 2006, p. xi).

2 We refer to L1 as the first language(s) of the child or any language(s) the child uses and understands with high levels of familiarity and proficiency, oftentimes called “mother tongue” (MT). We refer to L2/x as the second or later acquired language(s) of the child, that is, any language(s) they are learning and have emerging levels of familiarity and proficiency with.

3 According to CEDIL (2020), a mid‐range theory is between a project‐specific theory of change and grand theory. It is the theoretical underpinnings that allow us to make claims beyond one single project, but may not necessarily be universally applicable.

4 We follow the definition of systematic reviews and evidence syntheses from the Campbell Corporation: “a systematic review is an academic research paper… that uses a method called evidence synthesis to look for answers to a pre‐defined question.”

5 Bilingual or multilingual programs are defined as any that have more than one LOI, have additional language subject classes, or the students are bilingual or multilingual, or students are in mixed‐language/linguistically heterogenous classrooms.
