# Supplementary material for: Language of instruction in schools in low‐ and middle‐income countries: A systematic review
Source: Campbell Syst Rev. 2023 Oct 3;19(4):e1351. doi: 10.1002/cl2.1351 (PMC10546270; doi:10.1002/cl2.1351)

Additional Figures and Tables

Figure A.1. Full quantitative risk assessment tool with results


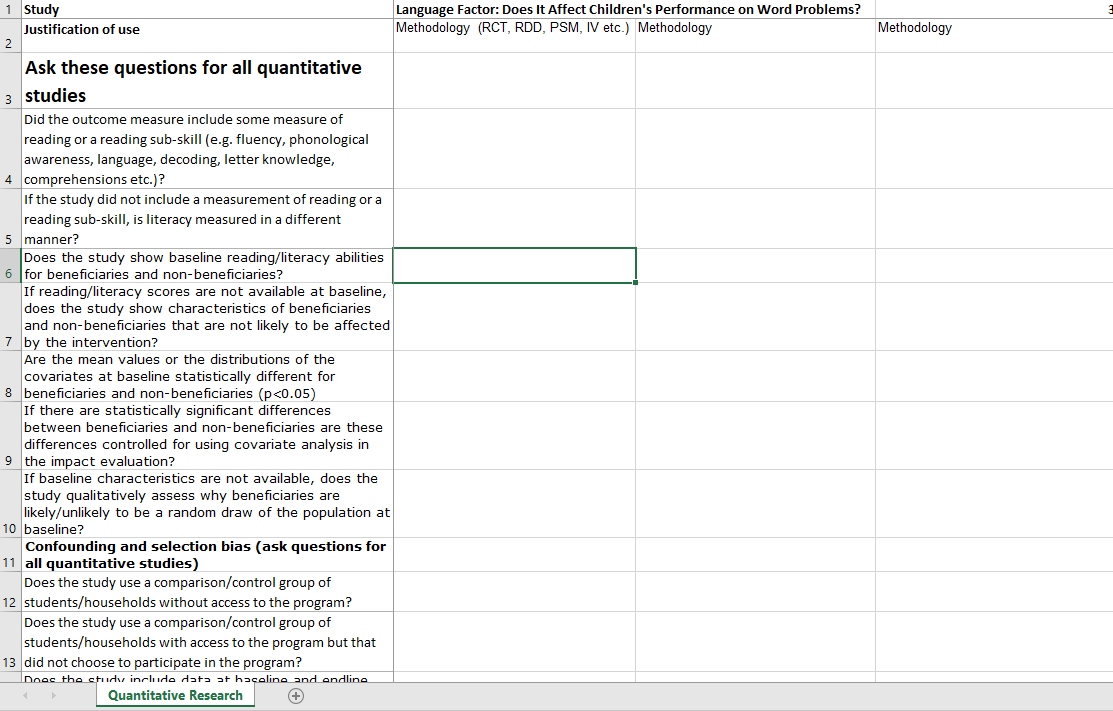


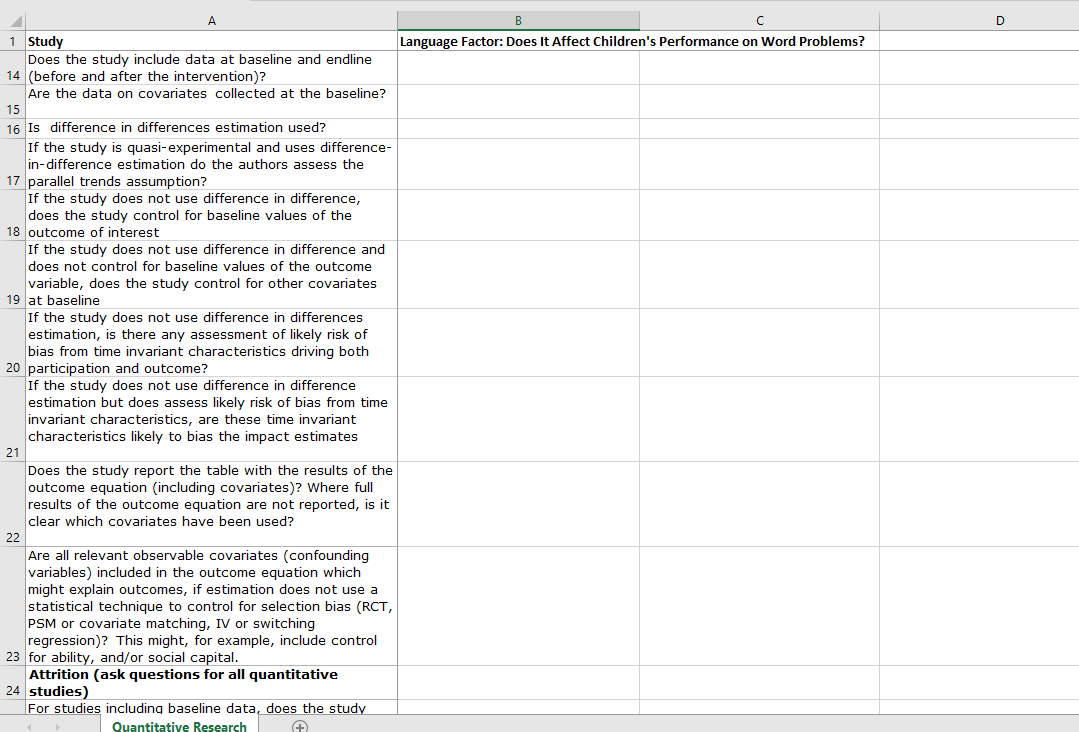

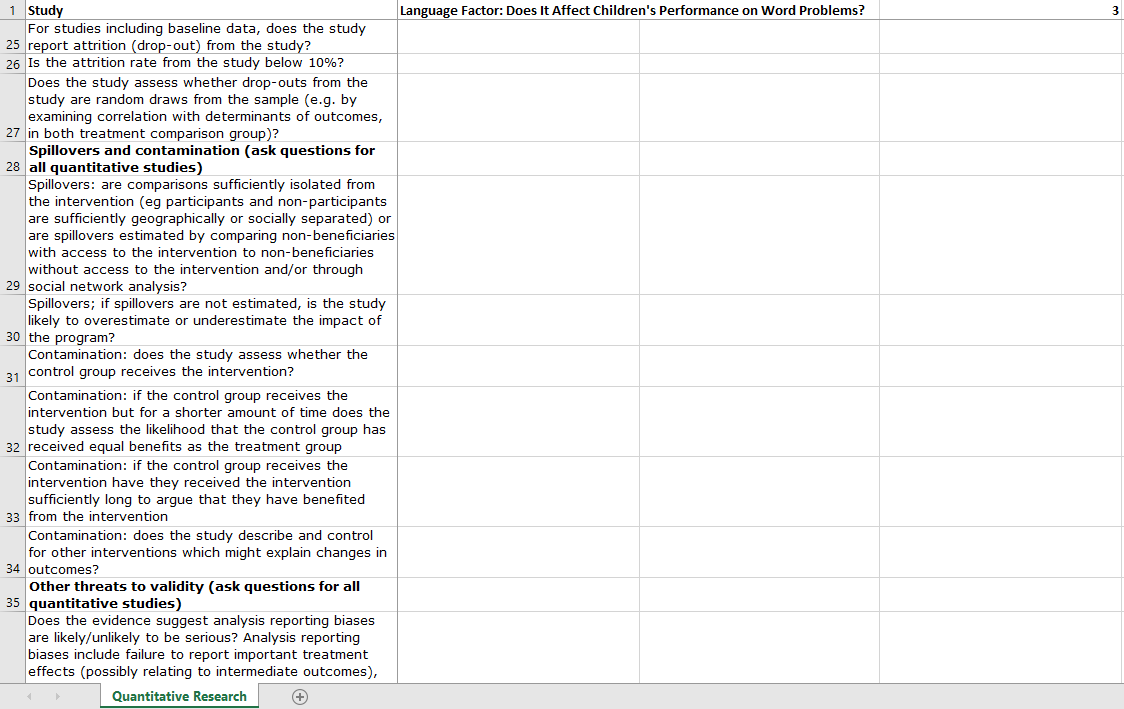

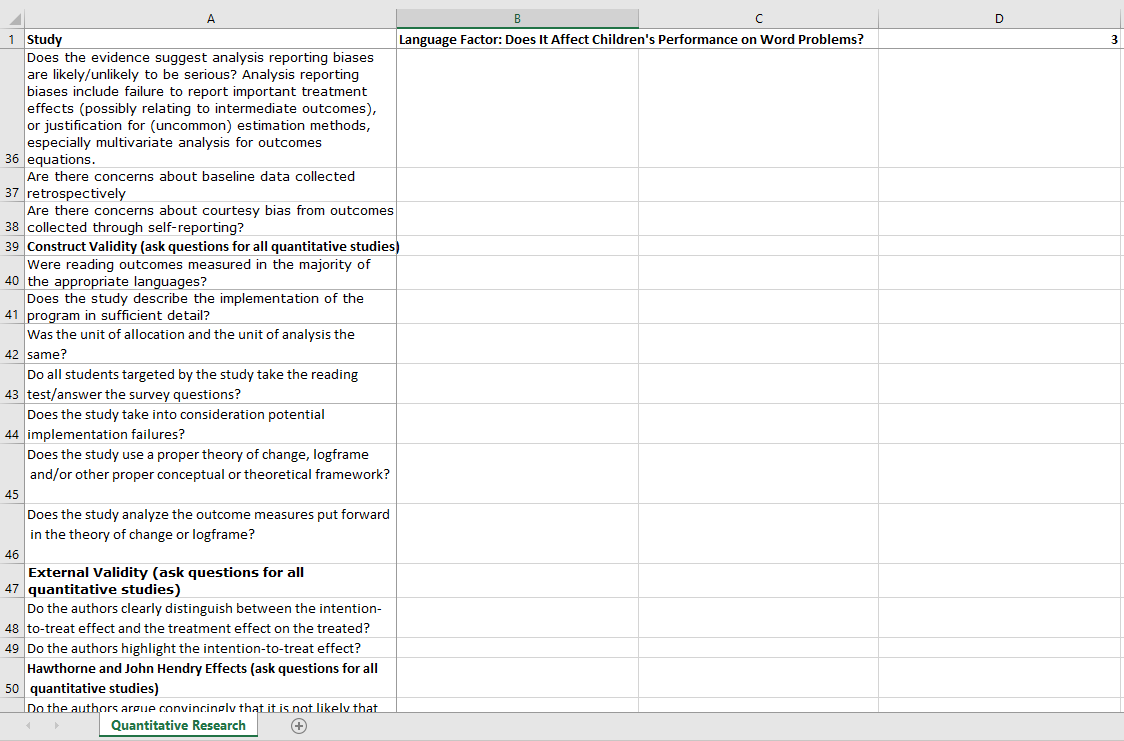

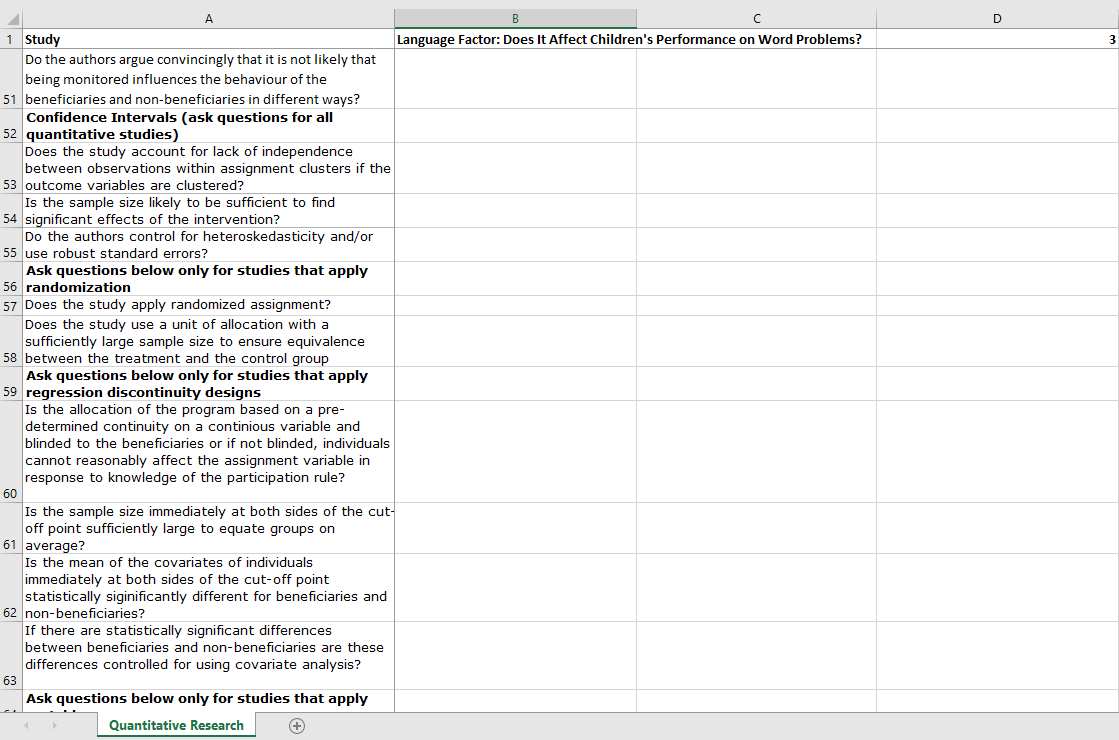

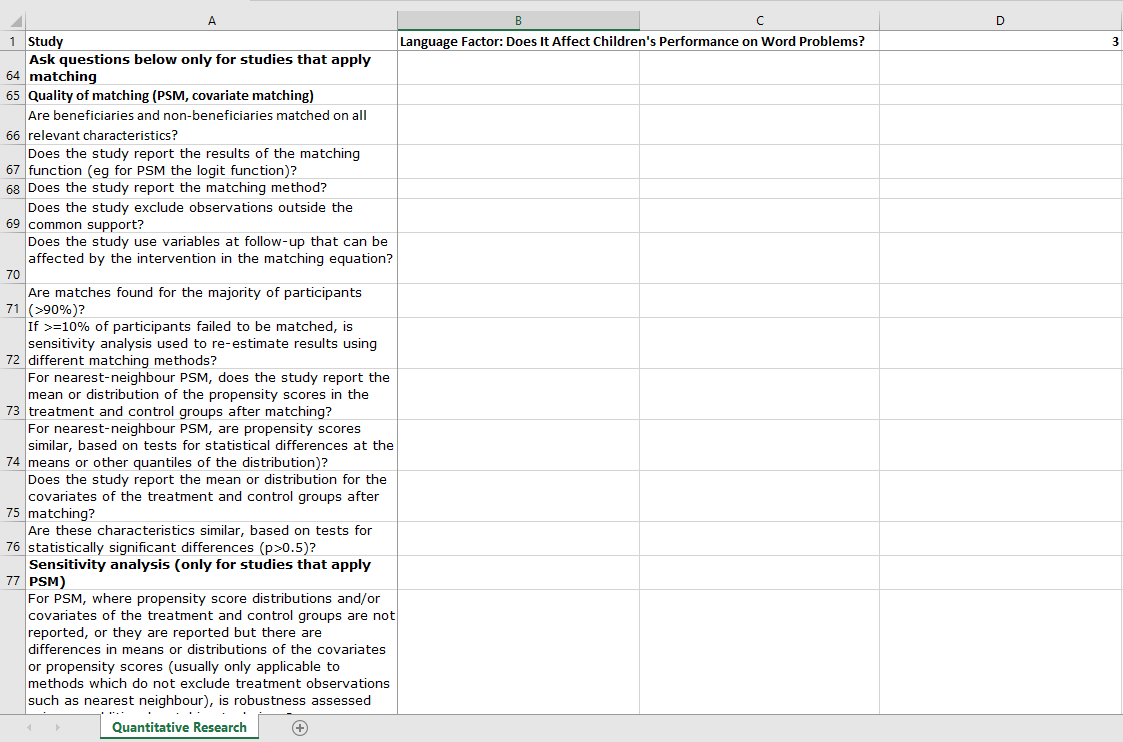

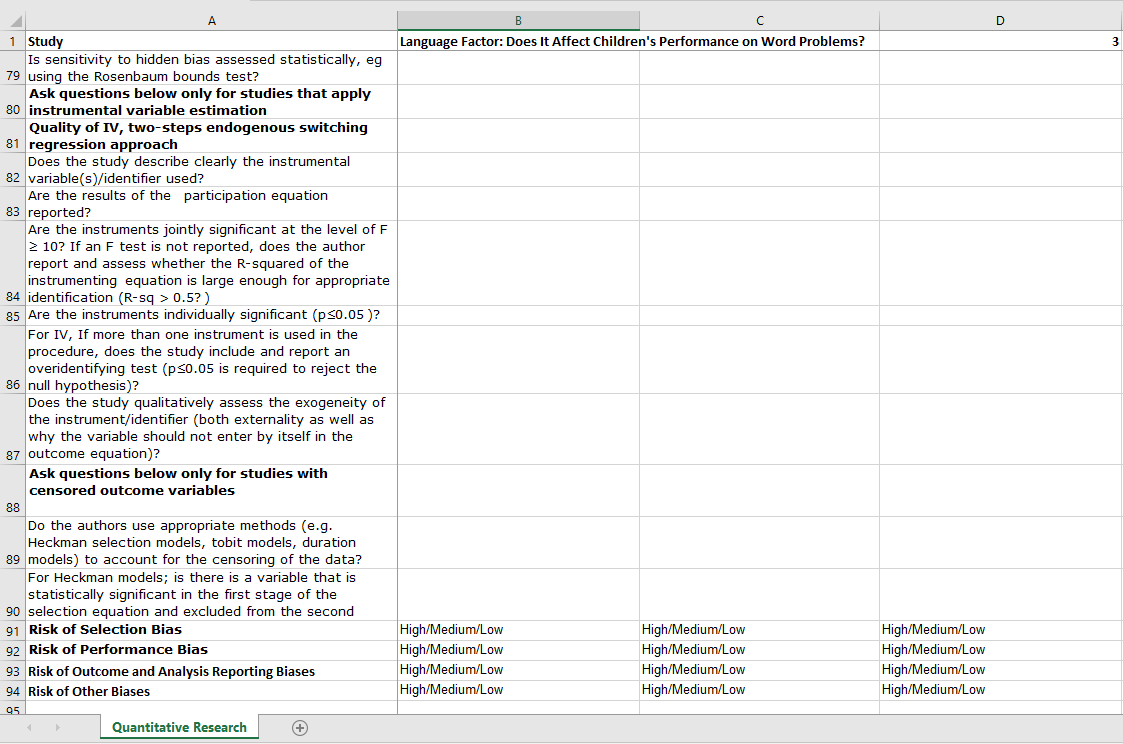


Figure A.2. Qualitative evidence review protocol


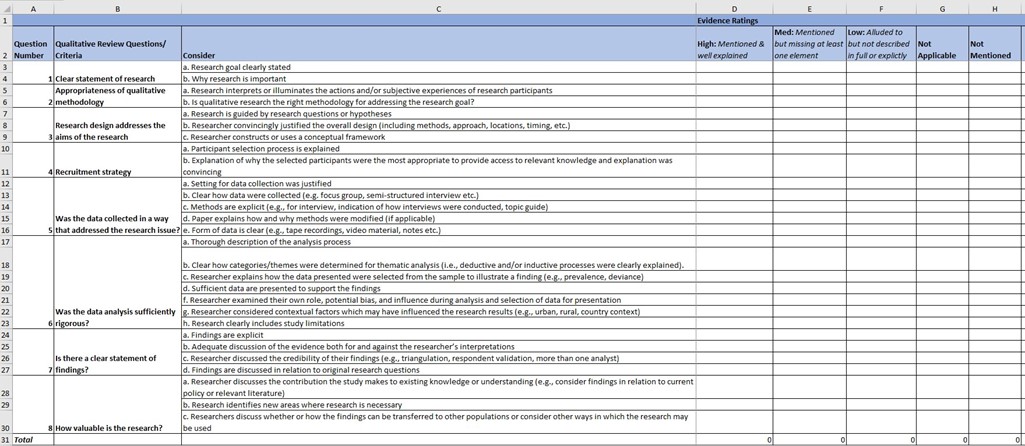


Figure A.3. Scoring Sheet to determine “high quality” studies from the included studies that used qualitative methodologies


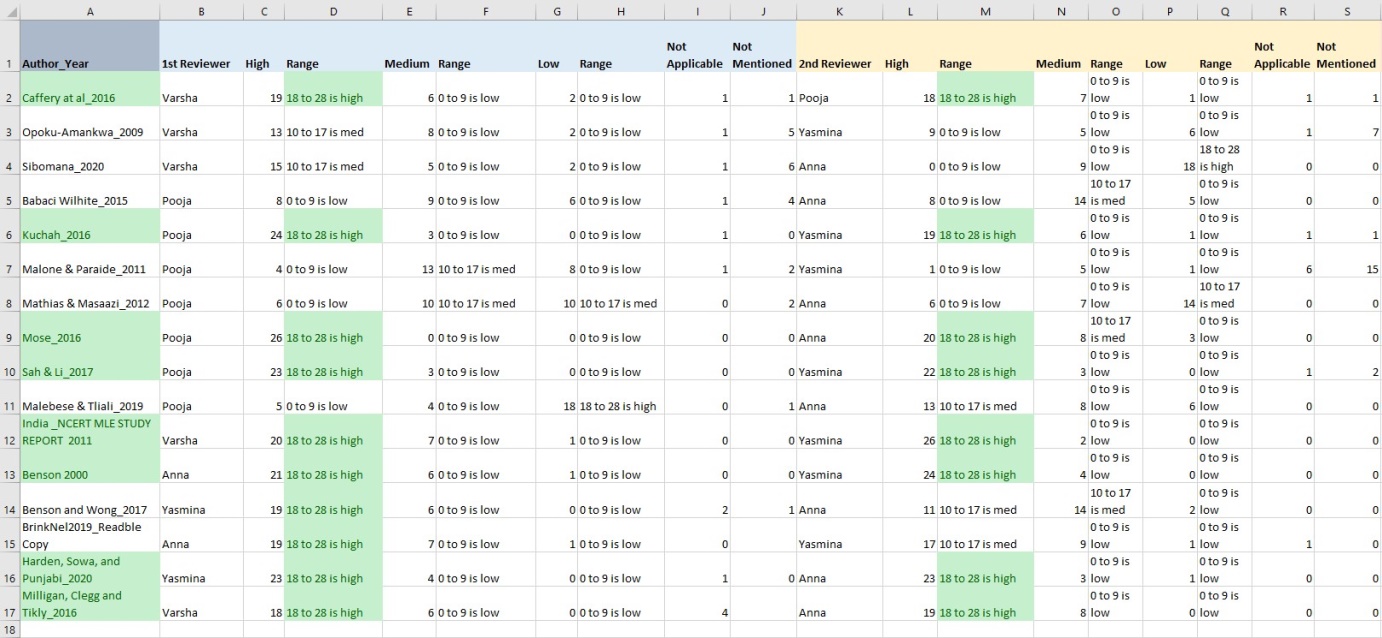

Supplement: Supplementary file 1 — Supporting information. [file CL2-19-e1351-s001.docx]
